# The antagonistic transcription factors, EspM and EspN, regulate the ESX-1 secretion system in *M. marinum*

**DOI:** 10.1128/mbio.03357-23

**Published:** 2024-03-06

**Authors:** Kathleen R. Nicholson, Rachel M. Cronin, Rebecca J. Prest, Aruna R. Menon, Yuwei Yang, Madeleine K. Jennisch, Matthew M. Champion, David M. Tobin, Patricia A. Champion

**Affiliations:** 1 Department of Biological Sciences, Eck Institute for Global Health, University of Notre Dame, Notre Dame, Indiana, USA; 2 Department of Molecular Genetics and Microbiology, Duke University School of Medicine, Durham, North Carolina, USA; 3 Department of Chemistry and Biochemistry, University of Notre Dame, Notre Dame, Indiana, USA; 4 Department of Immunology, Duke University School of Medicine, Durham, North Carolina, USA; Weill Cornell Medicine, New York, New York, USA

**Keywords:** *Mycobacterium*, type VII secretion, ESX-1, regulation, transcription

## Abstract

Bacterial pathogens use protein secretion systems to transport virulence factors and regulate gene expression. Among pathogenic mycobacteria, including *Mycobacterium tuberculosis* and *Mycobacterium marinum*, the ESAT-6 system 1 (ESX-1) secretion is crucial for host interaction. Secretion of protein substrates by the ESX-1 secretion system disrupts phagosomes, allowing mycobacteria cytoplasmic access during macrophage infections. Deletion or mutation of the ESX-1 system attenuates mycobacterial pathogens. Pathogenic mycobacteria respond to the presence or absence of the ESX-1 system in the cytoplasmic membrane by altering transcription. Under laboratory conditions, the EspM repressor and WhiB6 activator control transcription of specific ESX-1-responsive genes, including the ESX-1 substrate genes. However, deleting the *espM* or *whiB6* gene does not phenocopy the deletion of the ESX-1 substrate genes during macrophage infection by *M. marinum*. In this study, we identified EspN, a critical transcription factor whose activity is masked by the EspM repressor under laboratory conditions. In the absence of EspM, EspN activates transcription of *whiB6* and ESX-1 genes during both laboratory growth and macrophage infection. EspN is also independently required for *M. marinum* growth within and cytolysis of macrophages, similar to the ESX-1 genes, and for disease burden in a zebrafish larval model of infection. These findings suggest that EspN and EspM coordinate to counterbalance the regulation of the ESX-1 system and support mycobacterial pathogenesis.

**IMPORTANCE:**

Pathogenic mycobacteria, which are responsible for tuberculosis and other long-term diseases, use the ESX-1 system to transport proteins that control the host response to infection and promote bacterial survival. In this study, we identify an undescribed transcription factor that controls the expression of ESX-1 genes and is required for both macrophage and animal infection. However, this transcription factor is not the primary regulator of ESX-1 genes under standard laboratory conditions. These findings identify a critical transcription factor that likely controls expression of a major virulence pathway during infection, but whose effect is not detectable with standard laboratory strains and growth conditions.

## INTRODUCTION

Diverse pathogens rely on secretion systems to transport virulence factor substrates that promote bacterial survival in different host environments (1, 2). *Mycobacterium tuberculosis*, the causative agent of human tuberculosis, and *Mycobacterium marinum*, a poikilothermic fish pathogen, share a conserved (ESAT-6) secretion system [ESAT-6 system 1 (ESX-1)] that is required for pathogenesis (3–5). The ESX-1 system is composed of ESX core components (Ecc’s) that reside within the mycobacterial cell membrane (CM) and cytoplasm (4, 6–8). During macrophage infection, the ESX-1 system transports protein substrates that are secreted components of the ESX-1 machinery (9) and effectors that promote phagosomal damage (10–13). Phagosomal damage enables the essential interaction between pathogenic mycobacteria and the macrophage cytoplasm, which promotes macrophage death and cell-to-cell spread (12, 14–17).

In several Gram-negative pathogens, transcription is responsive to protein secretion systems (2, 18, 19). We and others have shown that the loss of the membrane-associated ESX-1 secretion system in *M. marinum* and *M. tuberculosis* resulted in widespread transcriptional changes (20–22). ESX-1 genes are a subset of the ESX-1 responsive genes. The gene most responsive to ESX-1 was *whiB6*. WhiB6 activates the transcription of ESX-1 substrate genes (20, 23, 24). EspM directly represses *whiB6* gene transcription in the absence of the ESX-1 components in the CM (20, 22). Reduced WhiB6 levels led to reduced ESX-1 substrate gene transcription, preventing accumulation of ESX-1 substrates in the absence of the transport machinery (20). Genetic deletion of the *espM* gene, which is divergently encoded from the *whiB6* gene, derepressed *whiB6* transcription and increased levels of ESX-1 substrates in *M. marinum* during standard laboratory growth conditions (22).

The deletion of ESX-1 substrate genes attenuates pathogenic mycobacteria in infection models (4, 25–28). Despite regulating the transcription of ESX-1 substrate genes, the deletion of *whiB6* or *espM* does not phenocopy the loss of ESX-1 substrate genes in macrophage models of infection (20, 22). Therefore, ESX-1 may be differentially regulated in the laboratory and during infection.

We previously used *lacZ*+ transcriptional fusions to measure EspM- and WhiB6-dependent transcription from the *M. marinum whiB6* promoter. β-Galactosidase activity was ~2-fold higher in the absence of both EspM and WhiB6 than in the absence of EspM alone (22), suggesting that *whiB6* transcription was activated in the absence of both EspM and WhiB6. We exploited our knowledge of the EspM repressor as a tool to identify an additional regulator of ESX-1 transcription.

## RESULTS

### EspN activates *whiB6* transcription in the absence of EspM

**Fig 1:**
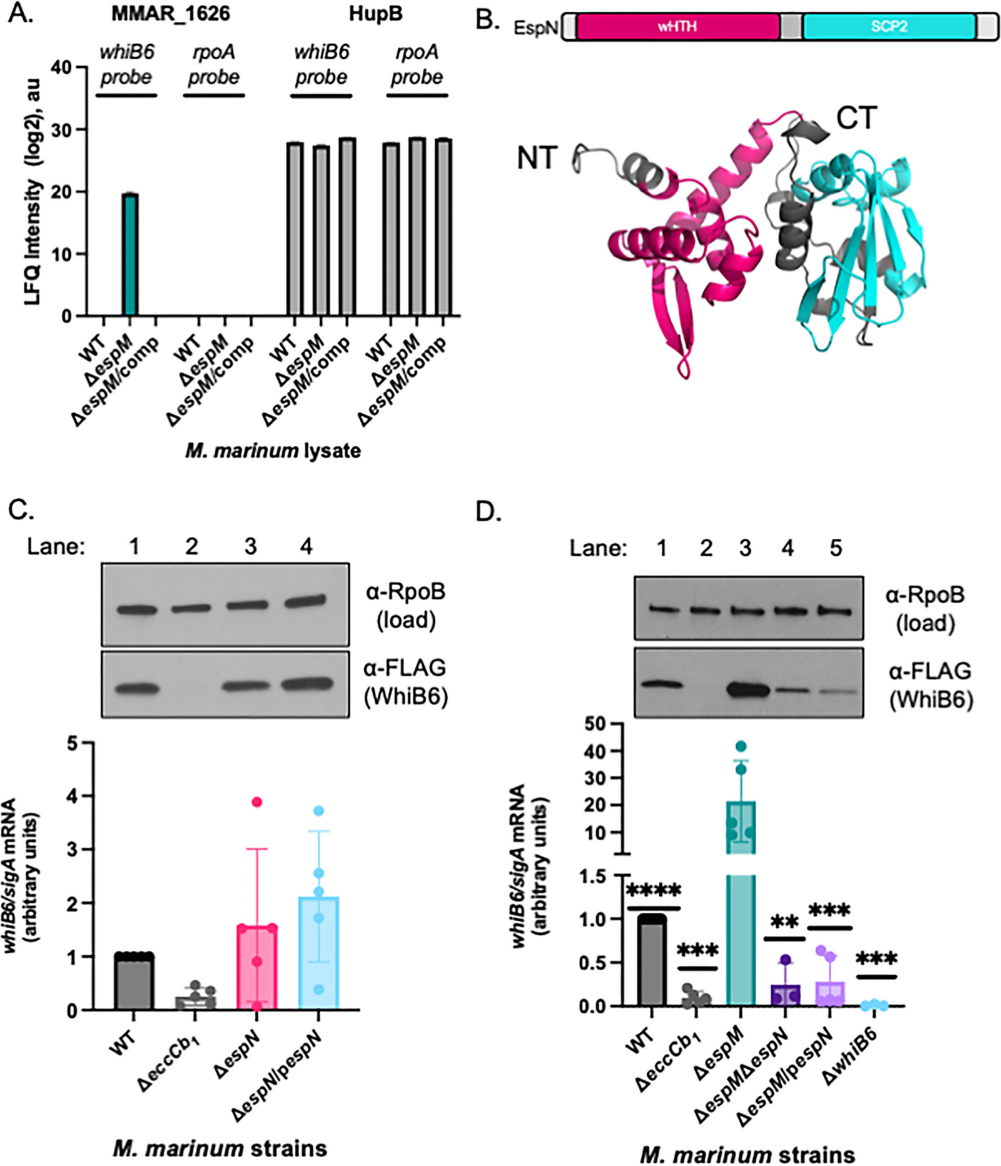
EspN binds the *whiB6* promoter and activates *whiB6* expression in the absence of EspM. (A) Mass spectrometry analysis of the DNA affinity chromatography showing enrichment of the MMAR_1626 and HupB proteins. The HupB protein binds non-specifically to both DNA probes. The scale represents the log2 intensity of Mass Spectral peak area (MS peak areas). The data were published in Sanchez et al. (22) and adapted in Data set S1. (**B)** The predicted domain structure of MMAR_1626, which we renamed EspN. Modeled using RoseTTAFold from Robetta (29). Model confidence: 0.80. (C) (Top) Western blot analysis of *M. marinum* cell-associated proteins. RpoB is a loading control. All strains include a *whiB6-3xFl* allele at the *whiB6* locus (20). (Bottom) Relative qRT-PCR analysis of *M. marinum* strains compared to *sigA* transcript levels. Statistical analysis was performed using one-way ANOVA (*P =* 0.0359*),* followed by a Dunnett’s multiple comparison test, which revealed no significant differences relative to the WT strain. (**D) **(Top) Western blot analysis of 10 µg of *M. marinum* whole-cell lysates. RpoB serves as a loading control. All strains include a *whiB6-3xFl* allele at the *whiB6* locus (20); (bottom) qRT-PCR of the *whiB6* transcript relative to *sigA*. Significance was determined using ordinary one-way ANOVA (*P* = 0.0001), followed by Tukey’s multiple comparisons test. Significance shown is relative to the Δ*espM* strain, with additional statistics of interest discussed in the text. ****P < 0.0001, ***P = 0.0002 for Δ*eccCb*_1_, **P = 0.0010, ***P = 0.0002 for Δ*espM*/p*espN*, ***P = 0.0009 for Δ*whiB6.* Western blots are representative of three independent biological replicates. All qRT-PCRs include at least three independent biological replicates, each in technical triplicate. ANOVA, analysis of variance; au, arbitrary units; qRT-PCR, quantitative reverse transcription PCR; SCP2, sterol carrier protein 2; wHTH, winged helix-turn-helix; WT, wild type.

To identify regulators activating *whiB6* transcription in the absence of EspM, we previously used the *whiB6-espM* intergenic region to enrich proteins from lysates from the *ΔespM M. marinum* strain (22). Mass spectrometry identified MMAR_1626 as the only protein enriched for binding the *whiB6-espM* intergenic region in the absence of *espM* [Fig. 1A; original data in Table S1 D of reference (22), analyzed again in SI Data set SIA through C].

MMAR_1626 is an unstudied putative transcriptional regulator with a predicted N-terminal winged helix-turn-helix (wHTH) domain, and a C-terminal sterol carrier protein 2 (SCP2) domain (Fig. 1B; Fig. S1D) (30, 31). Orthologs are found across *Mycobacterium* spp. and other high G + C Gram-positive bacteria, including members of the tuberculosis complex, *M. tuberculosis* (*Rv1725c*) and *M. bovis* (*Mb1754c*), and non-pathogens, including *M. smegmatis* (32, 33). Based on the following data, we propose naming this protein EspN, according to accepted nomenclature (34).

We hypothesized that EspN activates transcription of the *whiB6* gene. To test this hypothesis, we generated an unmarked *espN* deletion strain using allelic exchange (Fig. S1A and B) (35). The parental *M. marinum* M strain [wild type (WT)] has a *whiB6* allele with a C-terminal 3× FLAG epitope integrated at the *whiB6* locus (20). We generated the complementation strain by integrating a copy of the *espN* gene behind the constitutive mycobacterial optimal promoter at the *attL* site (Fig. S1C). We measured *espN* transcription using quantitative reverse transcription PCR (qRT-PCR) (Fig. S2A). The *espN* transcript was present in the WT strain but absent from the Δ*espN* strain (*P* = 0.0002, compared to the WT strain, inset). *espN* transcription was significantly increased (≥40-fold) in the Δ*espN*/*pespN* strain compared to the WT strain (*P* < 0.0114, Fig S2A).

To evaluate the role of EspN on *whiB6* transcription, we measured the *whiB6* transcript and protein levels in the Δ*espN* and Δ*espN*/*pespN* strains relative to the WT strain and Δ*eccCb*_1_ strains using qRT-PCR (Fig. 1C). We detected the *whiB6* transcript and protein in the WT strain (Fig. 1C, lane 1). The Δ*eccCb*_1_ strain does not produce the EccCb_1_ protein, a cytoplasmic component of the ESX-1 secretion system (4). The loss of EccCb_1_ destabilizes the ESX-1 membrane complex (7, 20, 36), resulting in transcriptional repression of *whiB6* (20, 22). Consequently, the WhiB6-Fl protein was not detected in the Δ*eccCb*_1_ strain (Fig. 1C, lane 2) (20). In contrast, *whiB6* transcription in the Δ*espN* and Δ*espN*/p*espN* strains was stochastic, with the mean from three experiments not significantly different from the WT strain. The levels of WhiB6-Fl protein in the Δ*espN* strain and the Δ*espN*/p*espN* strain were similar to that in the WT strain (Fig. 1C, lanes 3 and 4). We also tested the levels of *espF* and *esxA* transcription in the Δ*espN* strain and the Δ*espN*/p*espN* strain. EspF and EsxA are ESX-1 substrates that are both transcriptionally regulated by WhiB6 (20, 24). Similar to the *whiB6* transcript, the *espF* (Fig. S2B) and *esxA* (Fig. S2C) transcripts were stochastic. From these data, we conclude that *whiB6* transcription, and the transcription of *espF* and *esxA* was not dependent on EspN under the conditions tested.

We identified both EspM and EspN using DNA affinity chromatography with the *whiB6* promoter DNA (22). EspM was specifically enriched from the WT *M. marinum* lysate, while EspN was only specifically enriched from the Δ*espM* lysate [Fig. 1A; Table D in reference (22), and Data set SIA through C]. This led us to hypothesize that EspN regulated *whiB6* transcription in the absence of and in opposition to the EspM repressor. To test this, we deleted *espN* from the Δ*espM M. marinum* strain. We also introduced an integrating plasmid constitutively expressing *espN* into the Δ*espM* strain. Using qRT-PCR (Fig. S2D), we found that the *espN* transcript was absent from the Δ*espM*Δ*espN* strain and significantly increased in the Δ*espM*/*pespN* strain (~60- to 80-fold, *P* < 0.0001). We measured changes in the *whiB6* transcript and protein levels in the Δ*espM*Δ*espN* and Δ*espM*/p*espN* strains compared to the Δ*espM* strain (Fig. 1D). Consistent with our prior work, *whiB6* transcript was significantly reduced in the Δ*eccCb*_1_ strain (*P* < 0.0001) and significantly increased in the Δ*espM* strain relative to the WT strain (*P* < 0.0001) (20, 22). The WhiB6-Fl protein reflected the differences in transcript levels (Fig. 1D, lanes 1 through 3) (22). Deletion and overexpression of the *espN* gene in the Δ*espM* strain significantly reduced the *whiB6* transcript and WhiB6 protein levels (Fig. 1D, lanes 4 and 5) compared to the Δ*espM* (*P* = 0.0002 and *P* = 0.0009) and WT (*P* < 0.0001) strains. We conclude that *espN* overexpression in the Δ*espM* strain causes a loss of EspN function, similar to deletion of *espN* (37). Our data support that EspN is required for the elevated *whiB6* transcription in the Δ*espM* strain. Together, our findings suggest that EspN activates *whiB6* transcription in the absence of EspM, working in opposition to EspM at the *whiB6* promoter.

### EspN activates the transcription of ESX-1 component and substrate genes

**Fig 2:**
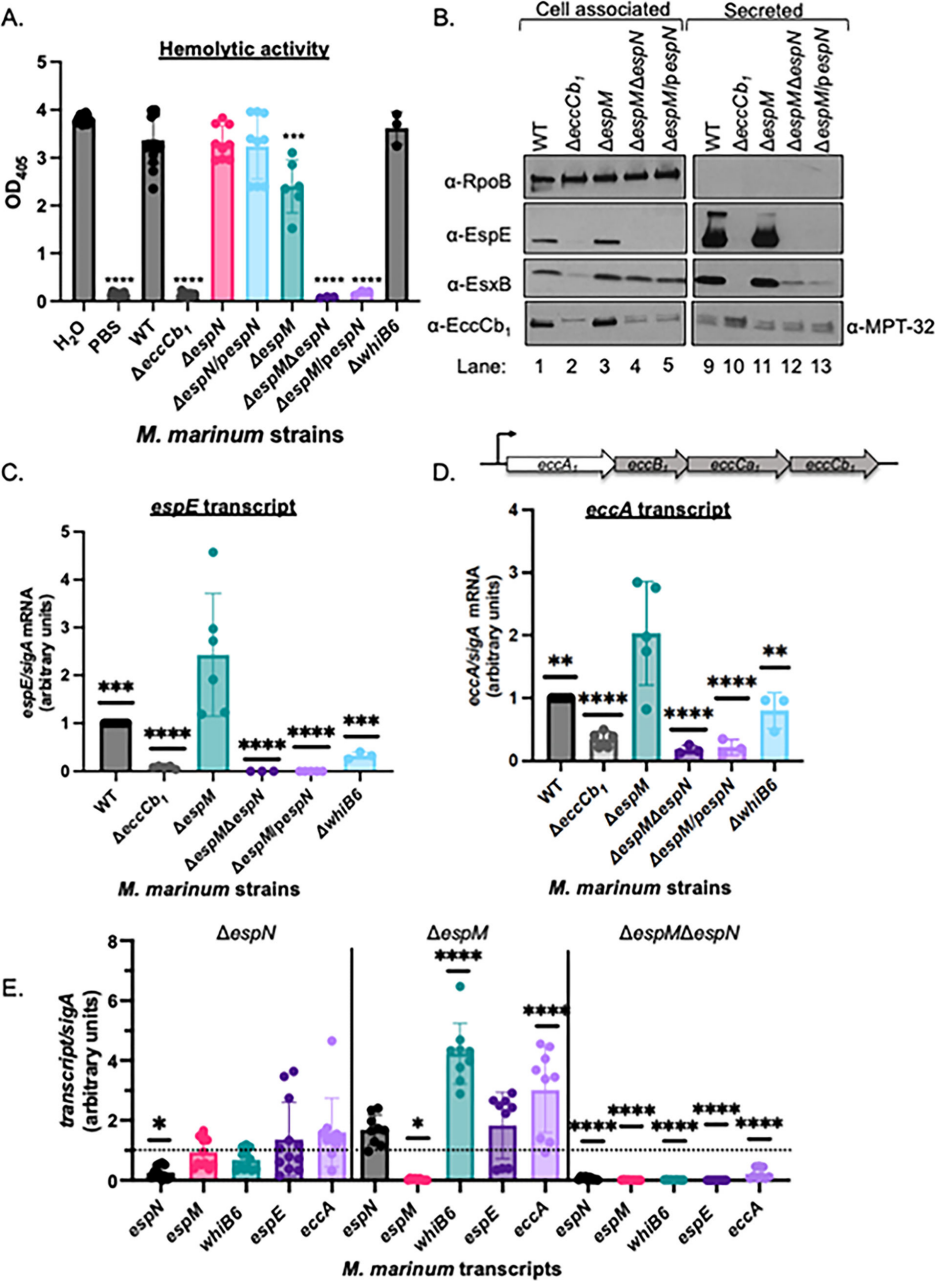
EspN and EspM control transcription of ESX-1 components and substrates. (A) Sheep red blood cell lysis measuring hemolytic activity of *M. marinum.* Statistical analysis was performed using ordinary one-way ANOVA followed by Dunnett’s multiple comparisons test relative to the WT strain. ****P < 0.0001, ***P = 0.0003. Data include three biological replicates each in technical triplicate. (**B)** Western blot of 10 µg of *M. marinum* cell-associated proteins. All strains include a *whiB6-Fl* allele. RpoB is a control for lysis. MPT-32 is a loading control for the secreted fractions. Blot is representative of three independent biological replicates. (**C)** Relative qRT analysis of the *espE* transcript compared to *sigA* transcript levels in *M. marinum*. Statistical analysis was performed using ordinary one-way ANOVA (*P* < 0.0001) followed by Tukey’s multiple comparisons test. Significance is shown relative to the Δ*espM* strain. ***P = 0.0010 (WT), ****P < 0.0001, ***P = 0.0003 (Δ*whiB6*). (**D)** Relative qRT analysis of the *eccA* transcript compared to *sigA* transcript levels in *M. marinum*. Statistical analysis was performed using ordinary one-way ANOVA (*P* < 0.0001) followed by Tukey’s multiple comparisons test. Significance shown relative to the Δ*espM* strain. **P = 0.0012 (WT), *P* = 0.0026 (Δ*whiB6*); ****P < 0.0001. (**E)** Relative qRT-PCR of the *espN*, *espM*, *whiB6*, *espE*, and *eccA* transcripts during macrophage infection. RAW 264.7 cells were infected with a multiplicity of infection of 20, and *M. marinum* strains were isolated at 4 hours post-infection. Outliers were identified using Robust regression and Outlier(ROUT) analysis, *Q* = 0.05%. Statistical analysis was performed using ordinary one-way ANOVA (*P* = 0.0004 for *ΔespN*, *P* < 0.0001 for Δ*espM* and Δ*espM*Δ*espN*) followed by Dunnett’s multiple comparisons test relative to the WT strain (dotted line) in each strain. For Δ*espN*, *P = 0.0352, Δ*espM*, *P = 0.0320, ****P < 0.0001; for Δ*espM*Δ*espN*, ****P < 0.0001. For all qRT-PCR, data include three independent biological replicates each in technical triplicate.

WhiB6 positively regulates the transcription of several ESX-1 substrate genes (20, 24). We therefore tested how regulation by EspM and EspN impacts ESX-1 function during laboratory growth. *M. marinum* exhibits contact-dependent, ESX-1-dependent hemolytic activity (5, 38). We measured sheep red blood cell (sRBC) lysis to define how EspM and EspN impact ESX-1 activity (Fig. 2A). Water and phosphate-buffered saline were “no bacteria” controls, causing total and baseline sRBC lysis as measured by OD_405_, respectively (Fig. 2A). *M. marinum* lysed sRBCs in an ESX-1-dependent manner (Δ*eccCb*_1_ vs WT, *P* < 0.0001). Deletion or overexpression of *espN* did not significantly impact hemolytic activity. The Δ*espM* strain lysed sRBCs slightly less than the WT strain (*P* = 0.0122). Although the loss of EspM or EspN alone did not greatly impact hemolytic activity, deletion or overexpression of *espN* in the Δ*espM* strain abolished hemolysis (*P* < 0.0001), similar to the Δ*eccCb*_1_ strain. These findings suggest that EspM and EspN are collectively required for ESX-1-dependent lytic activity. Moreover, EspN is essential for lytic activity only in the absence of EspM.

We sought to determine why EspM and EspN were essential for ESX-1 hemolytic activity. Considering that WhiB6 regulates ESX-1 transcription (20, 23, 24), if EspM and EspN were essential for lytic activity solely because they regulate *whiB6* transcription*,* then we would expect that the hemolytic activity of the Δ*espM*Δ*espN* strain would phenocopy the Δ*whiB6* strain. Consistent with our previous findings, the Δ*whiB6* strain retained hemolytic activity [Fig. 2A and reference (20)]. These data indicate that the loss of WhiB6 alone in the Δ*espM*Δ*espN* strain is insufficient to explain the loss of hemolytic activity.

To determine what caused the loss of hemolytic activity of the Δ*espM*Δ*espN* and Δ*espM*/p*espN* strains, we measured the production and secretion of two ESX-1 substrates, EsxB and EspE, using Western blot analysis. EsxB is an early substrate and likely secreted component of the ESX-1 system (4, 9). The loss of EsxB abolishes ESX-1 substrate secretion and hemolytic activity (5, 28). EspE, a late substrate, abolishes hemolytic activity but does not affect ESX-1 substrate secretion, except EspF (9, 28). EsxB and EspE were produced in (Fig. 2B, lane 1, α-EsxB and α-EspE) and secreted from (lane 9) the WT strain during *in vitro* growth. The Δ*eccCb*_1_ strain had reduced EsxB and EspE levels (lane 2) due to EspM-dependent repression of *whiB6* transcription (22). Neither protein was secreted (lane 10) due to a loss of the ESX-1 membrane components. EspE and EsxB were produced (lane 3) and secreted from the Δ*espM* strain (lane 11), consistent with our prior findings (22). Deletion or overexpression of the *espN* gene in the Δ*espM* strain abolished EspE production (lanes 4 and 5) and therefore secretion (lanes 12 and 13). EsxB was produced in both strains, but EsxB secretion was reduced compared to the WT and Δ*espM* strains (lanes 12 and 13).

Reduced EsxB secretion could be explained by reduced ESX-1 components in the CM. EccCb_1_ is required for the stability of the ESX-1 complex (20). As measured by Western blot analysis (Fig. 2B), the EccCb_1_ protein was detected in the cell-associated proteins from the WT strain and absent from the Δ*eccCb*_1_ strain (lanes 1 and 2, lower band). The Δ*espM* strain produced EccCb_1_ protein (lane 3,). In the absence of EspM and EspN, EccCb_1_ protein levels were lower than the WT strain (lanes 4 and 5).

The loss of EspE protein and reduced EccCb_1_ protein could be explained by reduced transcription. We tested if EspN regulated the transcription of *espE* or the ESX-1 component genes when EspM was absent. As measured by qRT-PCR (Fig. 2C), *eccCb*_1_ deletion significantly reduced (*P* < 0.0001) and *espM* deletion significantly increased (*P* = 0.0010) *espE* transcription relative to the WT strain, consistent with Fig. 2B and our previous findings (22, 39). Deletion or overexpression of *espN* in the Δ*espM* strain abolished *espE* transcription, significantly different from the Δ*espM* (*P* < 0.0001) and the WT strains (*P* < 0.0001). Importantly, *whiB6* deletion significantly reduced (*P* = 0.0003) but did not abolish the *espE* transcript. Therefore, the loss of WhiB6 was insufficient to cause the loss of *espE* transcription in the Δ*espM*Δ*espN* strain. We conclude that EspN is required for transcription of *espE* in the Δ*espM* strain (Fig. 2C).

The *eccCb*_1_ gene is downstream of three other ESX-1 component genes (Fig. 2D; *eccA*, *eccB*, and *eccCa*_1_*).* It is not known if the *ecc* genes are operonic. To test if reduced EccCb_1_ protein was due to reduced *ecc* transcription, we measured *eccA* transcription using qRT-PCR (Fig. 2D). The *eccA* transcript was significantly reduced (*P* < 0.0001) in the Δ*eccCb*_1_ strain and significantly increased (*P* = 0.0012) in the Δ*espM* strain as compared to the WT strain. Together, these data suggest that EspM represses *eccA* transcription. Deleting or overexpressing *espN* in the Δ*espM* strain significantly reduced *eccA* transcription compared to the Δ*espM* (*P* < 0.0001) and WT strains (*P* < 0.0001). Interestingly, while the *eccA* transcript in the Δ*whiB6* strain was significantly reduced compared to the Δ*espM* strain (*P =* 0.0026), it was not significantly different from the WT strain. These findings suggest that EspN activates and EspM represses *eccA* transcription independently of WhiB6.

To test how EspN and EspM impact transcription during infection, we infected RAW 264.7 macrophages with *M. marinum* strains lacking *espM*, *espN*, or both *espM* and *espN. M. marinum* can escape the phagosome between 2 and 4 hours post-infection [hpi (40)]. Since ESX-1 functions in the phagosome, we harvested the bacteria at 4 hpi and used qRT-PCR to measure *whiB6*, *espN*, *espM*, *espE*, and *eccA* transcripts in *M. marinum.* Transcription of *whiB6*, *espM*, *espE*, and *eccA* was not significantly different between the WT and Δ*espN* strains (Fig. 2E), in agreement with Fig. 1C. Similar to our findings during laboratory growth, deletion of *espM* significantly increased *whiB6* (*P* < 0.0001) and *eccA* (*P* < 0.0001) transcript levels relative to the WT strain. The *espN* and *espE* transcripts were also higher than the WT strain but did not reach significance. Strikingly, all five transcripts were significantly reduced in the Δ*espM*Δ*espN* strain compared to the WT strain. These data suggest that EspM and EspN jointly regulate *whiB6*, *espE*, and *eccA* transcription during macrophage infection under the conditions tested.

### EspN is required during *M. marinum* infection of macrophages and zebrafish

**Fig 3:**
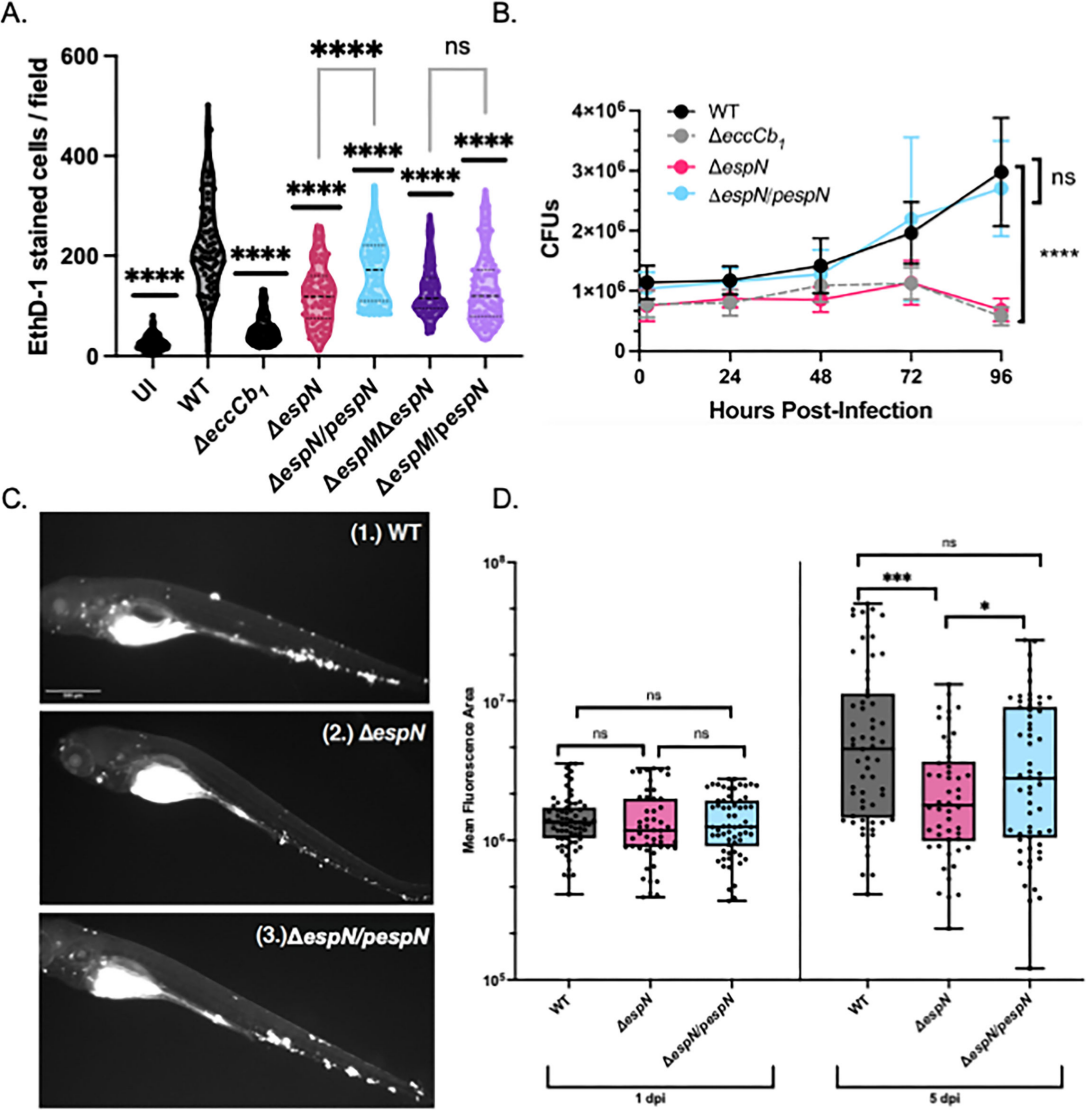
EspN is required for pathogenesis. (A) CFU of *M. marinum* strains (MOI = 0.2). The data points are the average of three independent biological replicates. Significance was determined using ordinary two-way ANOVA (*P* < 0.0001) followed by Tukey’s multiple comparisons test compared to the WT strain. The significance shown is compared to the WT strain at 96 hours post-infection (*P* < 0.0001). The Δ*eccCb*_1_ and Δ*espN* strains were also significantly different from the WT strain at 72 hpi (*P* = 0.0021 and *P* = 0.0024, respectively). (**B)** *M. marinum* burden in zebrafish infection measured using bacterial mCerulean fluorescence. Data are composed of two biological replicates with 20–30 independent infections per replicate. Statistical analyses were performed using one-way ANOVA followed by Tukey’s multiple comparisons of each group to the WT strain (***P = 0.00012, *P = 0.021). (**C)** Representative images of zebrafish infected with an initial dose of 150–200 fluorescent bacilli for (1.) WT, (2.) Δ*espN*, or (3.) Δ*espN*/p*espN* at 5 days post-infection. Scale bar is 500 µm. (**D)** Macrophage cytolysis as measured by EthD-1 staining 24 hours post-infection with *M. marinum* at an MOI of 4. Statistical analysis was performed using one-way ANOVA followed by Dunnett’s multiple comparisons test relative to the WT strain (****P < 0.0001). Each dot represents the number of EthD-1-stained cells in a single field. A total of 10 fields were counted using ImageJ for each well. Processing of three wells was performed for each biological replicate. A total of 90 fields were counted for each strain. CFU, colony-forming unit; ns, not significant.

Our data propose a model where EspM and EspN counterbalance to regulate ESX-1 gene transcription both in the laboratory and during infection. However, EspM obscures the regulatory role of EspN under laboratory conditions and during macrophage infection. *M. marinum* spp. cause macrophage cytolysis in an ESX-1-dependent manner. Strains lacking the ESX-1 system remain in the phagosome and are non-cytolytic (10). We infected RAW 264.7 cells with *M. marinum* (multiplicity of infection of 4) and imaged these 24 hpi using ethidium homodimer (EthD-1) staining to measure macrophage cytotoxicity (Fig. 3A). EthD-1 selectively stains DNA in cells with permeabilized cell membranes, reflecting the cytolytic activity of *M. marinum* (5, 41). Infection with WT *M. marinum* led to macrophage cytotoxicity (Fig. 3A). Uninfected and Δ*eccCb*_1_-infected macrophages exhibited significantly less cytotoxicity than WT-infected macrophages (*P* < 0.0001). Infection with the Δ*espN* strain significantly decreased macrophage cytotoxicity, similar to the Δ*eccCb*_1_ strain (*P* < 0.0001). Overexpressing *espN* in the Δ*espN* strain (*P* < 0.0001) partially complemented cytolytic activity. The Δ*espMΔespN* and Δ*espM*/p*espN* strains were non-cytolytic, similar to the *ΔespN* and *ΔeccCb*_1_ strains. From these data, we conclude that EspN is independently essential for *M. marinum* to cause macrophage cytotoxicity.

To test if *espN* was required for mycobacterial growth in macrophages, we performed CFU analysis (Fig. 3B). WT *M. marinum* grew within macrophages during infection. In contrast, the Δ*eccCb*_1_ strain was attenuated for growth in macrophages, resulting in CFUs significantly different from the WT strain at 72 and 96 hpi. Consistent with the cytotoxicity data, the Δ*espN* strain was attenuated for growth in the macrophages, similar to the Δ*eccCb*_1_ strain. Overexpression of the *espN* gene restored growth of *M. marinum* similar to the WT strain. From these data, we conclude that EspN is required for *M. marinum* growth and during macrophage infection.

To assess EspN’s importance during animal infection, we made constitutively fluorescent versions of the WT, *ΔespN*, and complemented *M. marinum* strains. Using the zebrafish larval model of mycobacterial infection, we monitored burden over 5 days of infection as measured by a validated fluorescence pixel count assay [Fig. 3C and D (42, 43)]. Bacterial burden was substantially reduced at 5 days post-infection. *In vivo*, we found that *ΔespN* had 3.4-fold reduced burden compared to WT at 5 days post-infection (*P* = 0.00012) and was complemented by constitutive expression of the *espN* gene (Fig. 3C and D). From these data, we conclude that EspN is required during zebrafish infection by *M. marinum.* Together, our data support that EspN is essential for mycobacterial infection.

### Overexpression of EspN causes loss of ESX-1 function

We found it curious that *espN* overexpression in the Δ*espM* strain phenocopied the Δ*espM*Δ*espN* strain during laboratory growth and macrophage infection. Our data argue against an unidentified mutation as the reason for the shared phenotypes. *espM* expression in the Δ*espM* strain complements all phenotypes associated with EspM loss. We performed whole-genome DNA sequencing on the WT, Δ*espM*, Δ*espN,* Δ*espM*Δ*espN*, and the Δ*espM*/p*espN* strains (Data set S2). No consistent mutations were identified between the Δ*espM*Δ*espN* and Δ*espM*/p*espN* strains that could account for the observed phenotypes.

**Fig 4:**
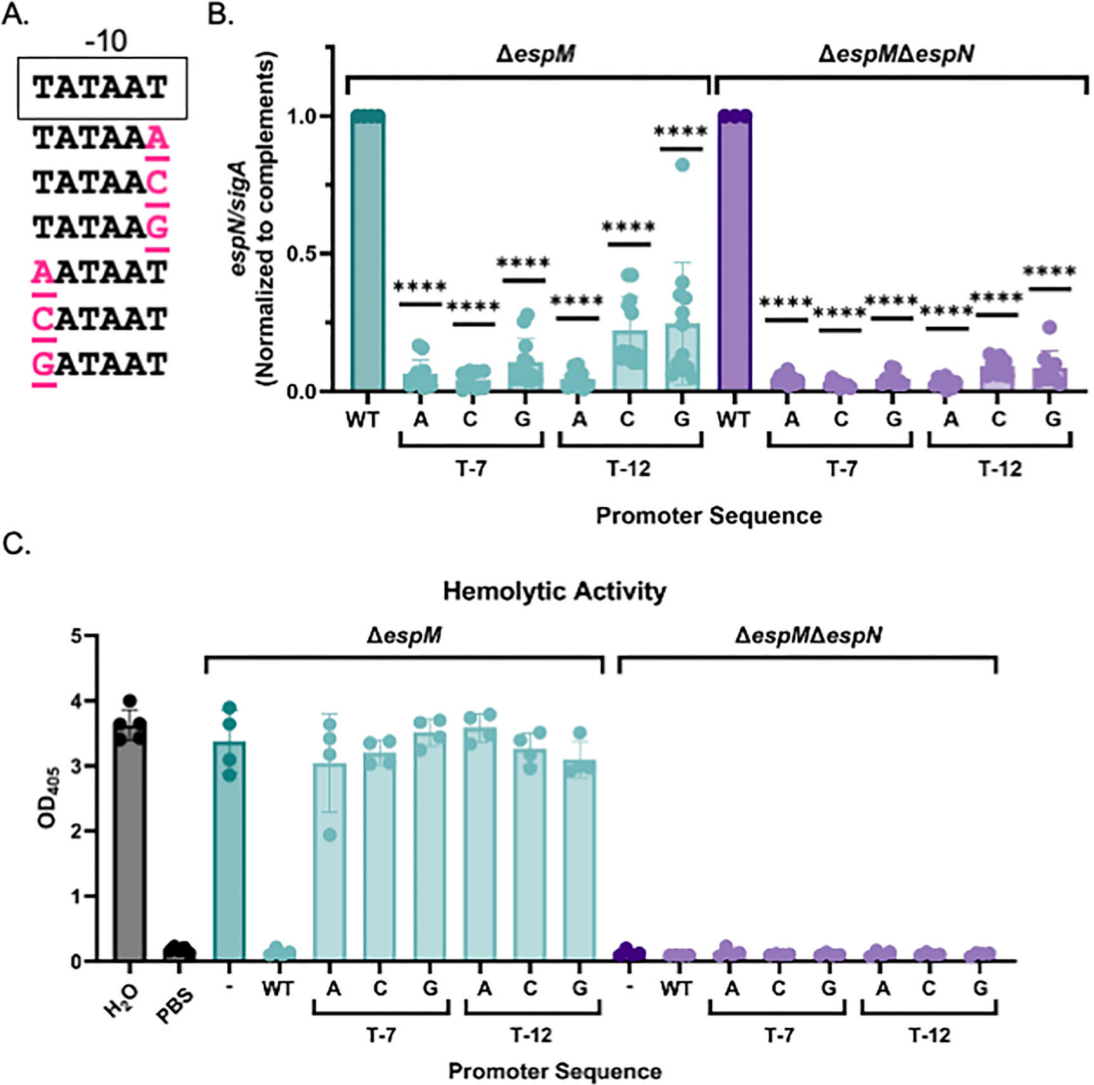
High levels of EspN transcription are required for dominance in the Δ*espM* strain. (A) Schematic of the −10 region from the mycobacterial optimal promoter driving *espN* transcription. Pink residues are mutations in the −7 and −12 positions. (**B)** Relative qRT analysis of the *espN* transcript compared to *sigA* transcript levels in *M. marinum*. Outliers were identified using ROUT analysis, *Q* = 0.5%. Statistical analysis was performed using ordinary one-way ANOVA (*P* < 0.0001) followed by Dunnett’s multiple comparisons test. Significance is shown relative to the Δ*espM/pespN* or Δ*espM*Δ*espN*/p*espN* strain. ****P < 0.0001. Data include three biological replicates each in technical triplicate. (**C)** sRBC lysis measuring hemolytic activity of *M. marinum.* Statistical analysis was performed using ordinary one-way ANOVA followed by Dunnett’s multiple comparisons test relative to the Δ*espM* or Δ*espM*Δ*espN* strain. ****P < 0.0001. Data include three biological replicates each in technical triplicate.

We hypothesized that *espN* overexpression was affecting endogenous EspN activity. We designed mutations in the mycobacterial optimal promoter focusing on −7 and −12 positions (Fig. 4A). We confirmed the mutations by DNA sequencing, introduced the p*espN* plasmids into the Δ*espM* or *ΔespMΔespN M. marinum* strains, and measured *espN* expression using qRT-PCR. The mutated plasmids resulted in significantly reduced *espN* expression compared to the parental plasmid (Fig. 4B; WT, *P* < 0.0001). Although overexpression of *espN* abolished the hemolytic activity of the Δ*espM* strain (*P* < 0.0001), reduced *espN* expression did not significantly alter hemolysis of the Δ*espM* strain (Fig. 4C). From these data, we conclude that the *espN* overexpression caused the loss of hemolysis in the Δ*espM* strain. Notably, reduced *espN* expression from the mutated promoter did not restore hemolysis of the Δ*espM*Δ*espN* strain (Fig. 4C).

### EspE and the N-terminus of EspM are linked to EspN function

**Fig 5:**
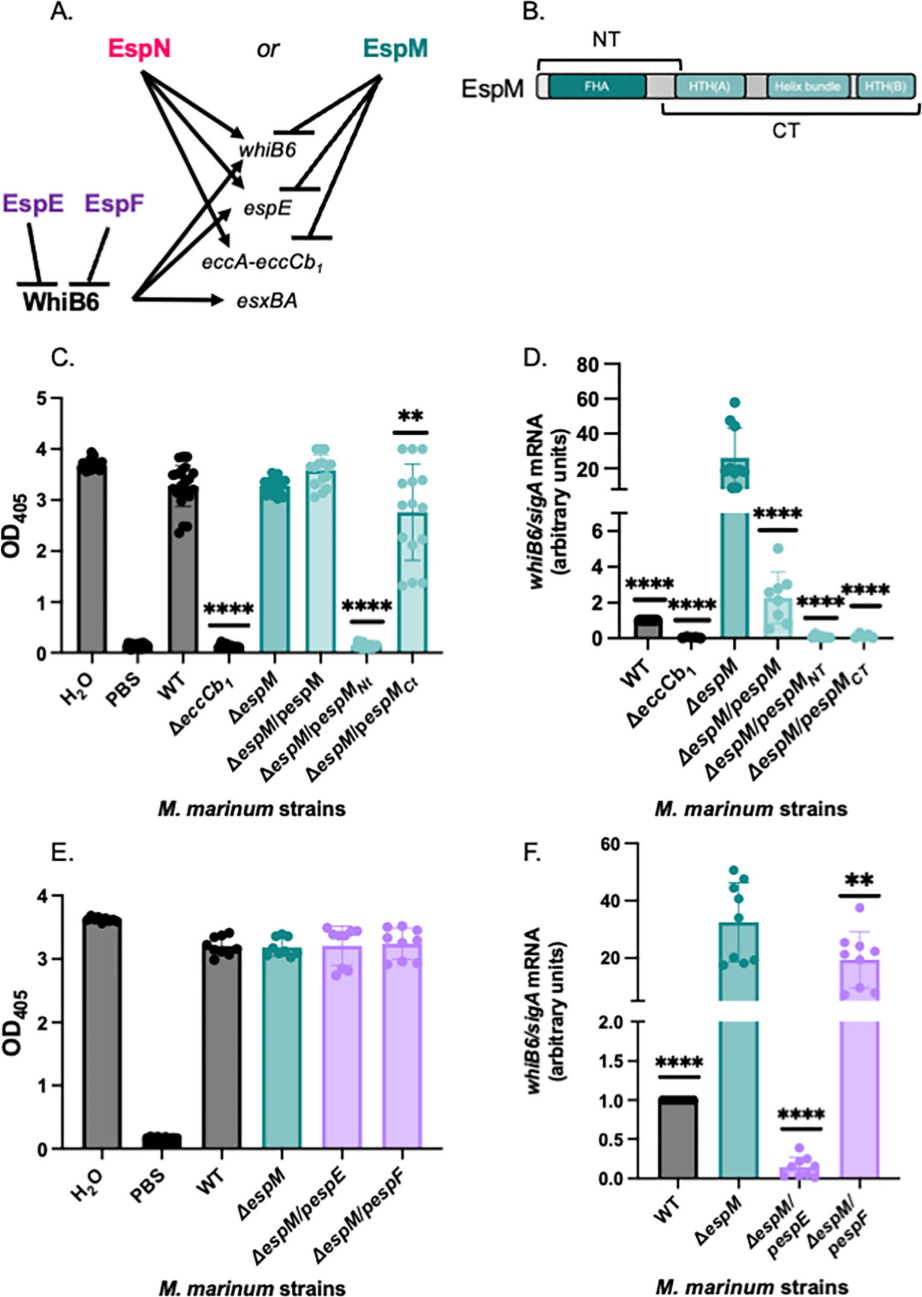
Overexpression of the EspM N-terminus or EspE negatively impacts ESX-1 transcription in the absence of EspM. (A) Schematic of transcriptional regulation by EspM, EspN, and WhiB6. EspE and EspF are ESX-1 substrates that negatively regulate the WhiB6 transcription factor (28). (**B)** Schematic of the predicted domains of the EspM protein. (**C)** sRBC lysis measuring the hemolytic activity of *M. marinum.* Outliers were identified using ROUT analysis, *Q* = 0.05%. Statistical analysis was performed using one-way ANOVA (*P* < 0.0001) followed by Dunnett’s multiple comparisons test (**P = 0.0034, ****P < 0.0001). The data include at least three independent biological replicates each in technical triplicate. (**D)** Relative qRT-PCR analysis of *whiB6* compared to *sigA* transcript levels. Significance was determined using ordinary one-way ANOVA (*P* < 0.0001), followed by Dunnett's multiple comparisons test (****P < 0.0001) relative to the Δ*espM* strain. The qRT-PCR data include at least three independent biological replicates each in technical triplicate. (**E)** sRBC lysis measuring hemolytic activity of *M. marinum.* Outliers were identified using ROUT analysis, *Q* = 0.05%. Statistical analysis was performed using ordinary one-way ANOVA (*P* = 0.9639), which did not indicate significant differences. The data include at least three independent biological replicates each in technical triplicate.** (F) **Relative qRT-PCR analysis of *whiB6* compared to *sigA* transcript levels. Outliers were identified using ROUT analysis (*Q* = 0.5%). Significance was determined using ordinary one-way ANOVA (*P* < 0.0001), followed by Dunnett's multiple comparisons test (**P = 0.0070, ****P < 0.0001) relative to the Δ*espM* strain. The qRT-PCR data include at least three independent biological replicates each in technical triplicate. FHA, forkhead-associated domain; HTH, helix-turn-helix; NT, N-terminus.

To understand the transcriptional network (Fig. 5A), we examined regulatory interactions between EspM and EspN. Assessing *espN* transcription in the Δ*espM* strain and *espM* transcription in the Δ*espN* strain (Fig. S4A and B) revealed no significant changes. We conclude that EspM and EspN do not regulate each other transcriptionally under the conditions tested.

Because EspN overexpression abolished hemolytic activity and ESX-1 gene expression in the Δ*espM* strain, we asked if overexpressing other regulators impacted ESX-1 function in the absence of EspM. EspM has an N-terminal forkhead-associated (FHA) domain (EspM_NT_, Fig. 5B) and two HTH domains between a helical bundle (EspM_CT_ in Fig. 5B) at the C-terminus. We constitutively expressed the *espM*, *espM_NT_*, and *espM_CT_* genes including a C-terminal V5 epitope tag in the Δ*espM* strain. All EspM-V5 proteins were expressed in *M. marinum* (Fig. S5A, lanes 4–6). *espM-V5* expression in the Δ*espM* strain did not significantly impact hemolytic activity (Fig. 5C). *espM_NT_-V5* expression abrogated hemolytic activity of the Δ*espM* strain (*P <* 0.0001). *espM_CT_-V5* expression in the Δ*espM* strain significantly impacted hemolytic activity (*P =* 0.0034), causing stochasticity relative to the other strains. These data suggested that the Δ*espM*/p*espM_NT_*-V5 strain effectively phenocopied the Δ*espM*Δ*espN* strain regarding ESX-1-dependent hemolytic activity (Fig. 3B).

Overexpressing the EspM-V5 or the EspM_CT_-V5 protein significantly reduced the *whiB6* (*P* = 0.0014, *P* = 0.0050; Fig. 5C), *eccA*, and *espE* transcripts (Fig. S5B through D) relative to the Δ*espM* strain. Overexpression of EspM-V5 resulted in reduced WhiB6Fl, EccCb_1_, EspE, and EsxB protein levels compared to the Δ*espM* strain (Fig. S5A). Expression of the *espM_CT_* gene resulted in a loss of detectable WhiB6-Fl protein and further reductions of the EccCb_1_, EspE and EsxB proteins compared to the Δ*espM* and Δ*espM*/p*espMV5* strains (Fig. S5A). The EspM_CT_ binds the *whiB6* promoter and represses *whiB6* transcription in *M. marinum* (22, 44). Although EspM_NT_ does not bind the *whiB6* promoter (22), expression of *espM_NT_* in the Δ*espM* strain significantly reduced *whiB6* (*P* = 0.0015), *espE* (*P* < 0.0001), and *eccA* (*P* < 0.0001) transcription, resulting in a loss of EspE and reduced WhiB6-Fl and EccCb_1_ proteins compared to the Δ*espM* and complemented strains (Fig. 5C; Fig. S5A; statistical analyses in Fig. S5). None of the strains tested exhibited significant reductions in *espN* transcription (Fig. S5E).

EspE and EspF are dual-functioning ESX-1 substrates. Both proteins negatively regulate WhiB6 activity in *M. marinum*, and their secretion is required for hemolytic activity and for virulence (28). Overexpressing *espE* or *espF* did not impact hemolytic activity (Fig. 4E) but significantly reduced *whiB6* transcription in the Δ*espM* strain (Fig. 4F). However, while *espE* overexpression reduced *whiB6* transcription to levels below the WT strain, overexpression of *espF* resulted in transcription levels ~20× higher than the WT strain. From these data, we conclude that overexpression of EspE and EspM_NT_, but not EspF, results in phenotypes consistent with EspN loss of function in the absence of EspM.

## DISCUSSION

Our prior studies hinted at the existence of an additional ESX-1 activator (22). Here, we discovered and characterized EspN, a transcriptional regulator of the ESX-1 system that is essential for infection. Our data suggest that EspN and EspM function as a switch that regulates the transcription of ESX-1 genes. When the ESX-1 system is absent, EspM represses *whiB6*, ESX-1 component (EccA and others), and substrate genes, preventing substrate accumulation in the absence of secretion (20, 22). EspM also regulates the transcription of additional genes in *M. marinum* (22). In the presence of the ESX-1 system, WhiB6 activates ESX-1 substrate gene transcription and other genes, allowing production of substrates during active secretion (20, 23, 24). The presence of EspM masked a role for EspN in regulating ESX-1 gene transcription. However, in the absence of EspM, EspN is essential for ESX-1 activity likely because it regulates *espE* transcription and contributes to *eccA* transcription, optimizing ESX-1 component production and substrate secretion. Deleting *espN* attenuated the cytolytic activity and growth of *M. marinum* during macrophage infection, similar to a loss of ESX-1. EspN was likewise necessary for robust infection in zebrafish larvae. Our findings suggest that EspN is required for ESX-1 function during infection possibly due to transcriptional regulation of ESX-1 genes. Deleting both *espM* and *espN* from *M. marinum* abolished cytolytic activity and transcription of *whiB6*, *espE*, and *eccA* during macrophage infection, which differed from deleting *espN* alone. Finally, overexpression of *espN*, *espM_NT_*, and *espE* specifically disrupted ESX-1 regulation, demonstrating connections in the transcriptional network. Together, these data demonstrate a critical role for EspN and the ESX-1 transcriptional network during infection.

Our data suggest that EspN may function both independently and together with EspM to regulate transcription and to promote pathogenesis. *espN* deletion did not have regulatory phenotypes under laboratory conditions or during macrophage infection under the conditions we tested. However, the Δ*espN* strain was attenuated both in macrophage and in zebrafish larvae. EspN-dependent regulation of ESX-1 or additional genes may be essential for virulence. The deletion of both EspN and EspM abolished ESX-1 transcription under laboratory conditions and during macrophage infection, which differed from deleting either regulator alone. Defining the regulatory targets of EspN, as well as how the EspM and EspN regulons compare, will likely require measuring global gene expression during specific stages of infection.

Some of our strains exhibited variable transcript levels (the Δ*espN* and Δ*espM* strains; Fig. 1C, D, 2D, E, 5C and D) or hemolytic activity (Δ*espM*, Fig. 2A; Δ*espM*/p*espM_CT_*, Fig. 5F) across multiple experiments. Our studies relied on population averages for each assay. In regulatory systems such as Type III secretion-dependent transcriptional regulation in *P. aeruginosa*, persister cells in *Escherichia coli*, and cannibalism and competence in *B. subtilis*, stochasticity reflects that individual cells within a population have different gene expression patterns (45, 46). This “bistability” indicates a switch between two states rather than intermediate ones (45). The stochasticity of our results may suggest that ESX-1 regulatory network includes a bistable switch that operates early during infection, possibly through pairs of mutually exclusive regulators or positive autoregulation (45). While the molecular nature of the switch is unknown, EspM and EspN likely cannot simultaneously occupy the *whiB6*, *eccA*, or *espE* promoters. Host-specific signals may regulate the switch, including oxidative stress, pH, and other phagosomal cues. *espN* is divergently transcribed from a putative methyltransferase gene (*MMAR_1627*) (32). We do not know yet if the genes surrounding *espN* are important for regulation of ESX-1; methylation has been shown to control bacterial genetic switches (47, 48).

ESX-1 expression varies in different strains under laboratory conditions (24). Consistent with our model, ESX-1 gene expression is upregulated in the host (49), and transcriptomic studies in *M. marinum* suggested differential regulation of virulence genes in a variety of environments (50). The differential regulation of protein secretion systems under laboratory conditions and in the host is a common theme in protein secretion in bacterial pathogens. For example, some clinical *Vibrio cholerae* strains have active Type VI systems under laboratory conditions, while the pandemic strains only activate their Type VI systems in the host (51). The specialized Type III secretion systems in *Vibrio* species are regulated by bile salts (52) and by Ca^2+^ and host cell contact in *Pseudomonas* (53–55).

Our findings suggest that EspN activity or expression might respond to host-specific signals. Notably, transcriptional profiling of *M. tuberculosis* from infected macrophages isolated from mouse infections revealed significant upregulation of the *espN* (Rv1725c) transcript compared to broth-grown culture (56). At 1 week post-infection in a mouse intravenous model, a TnSeq screen identified a ~2-fold decrease in representation of transposon insertions in *M. tuberculosis Rv1725c* [*P* = 0.0039 (57)]. However, at 4 and 8 weeks, this effect was no longer present (57, 58). Together, these data suggest that complex regulatory circuits and genetic requirements operate at discrete stages of infection. EspN’s C-terminal SCP2 domain (Fig. 1B) could differentially localize the protein in the mycobacterial cell in response to the host environment (59, 60) to mediate interaction with the mycobacterial cell membrane under specific conditions. SCP2 domains also mediate lipid transfer of sterols such as cholesterol and fatty acids (61, 62), which are an energy source for mycobacterial pathogens during infection (63, 64). Focusing on EspN’s SCP2 domain may reveal how EspN senses and responds to the host environment.

Gene dosage is important for biological function across organisms. Increases in copy number or expression of wild-type genes can cause mutant phenotypes, such as aggregation or mislocalization (37). We were surprised that the overexpression of *espN*, *espM_NT_*, and *espE* in the absence of *espM* resulted in the same regulatory phenotypes in *M. marinum* as deleting *espN* from the Δ*espM* strain*. espN*, *espM_NT_*, or *espE* overexpression could result in EspN aggregation or mislocalization. Transcription factor multimerization is a regulatory mechanism in bacterial gene expression (65, 66). In higher organisms, transcription factors can form aggregate-like bodies that serve as functional regulatory mechanisms which mimic loss of function (67). EspN may form multimeric aggregates that prevent DNA interaction. Alternatively, EspN may be mislocalized under overexpression conditions. The EspM_NT_ is a predicted FHA domain. Proteins with FHA domains regulate other Gram-negative secretion systems post-transcriptionally (68–70). Our data also suggest that EspM may be processed *in vitro*. EspM cleavage might remove it from the promoter, similar to the cI repressor from the λ phage (65). Alternatively, cleavage may liberate the N-terminus to regulate EspN activity, allowing the EspM_CT_ to bind DNA and repress gene expression.

Our studies were limited by an inability to complement *espN* expression in the Δ*espM*Δ*espN* strain. We tested overexpression, endogenous, and inducible promoters to restore *espN* transcription in the Δ*espM*Δ*espN* strain. We were unable to restore transcription to the levels of the Δ*espM* strain. We suspect that architecture or chromosomal location of *espN* may be important for proper regulation and expression.

Several additional transcription factors regulate *whiB6* and the *espACD* operon, which encodes secreted substrates essential for ESX-1 secretion in *M. tuberculosis* (25, 71–73). PhoPR and MprAB regulate *whiB6* expression directly and through the EspR regulator (74–78), in response to pH or stress, both of which are important for survival in the acidified phagosome (79–81). However, the *espACD* operon is dispensable for ESX-1 secretion and pathogenesis in *M. marinum*, and regulation of *whiB6* by PhoPR has not been reported (9, 82). Although this may suggest divergence in ESX-1 regulation between *M. marinum* and in *M. tuberculosis*, the ESX-1 transcriptional network is conserved (20, 21, 83). Widespread ESX-1-dependent gene expression has been reported in both mycobacterial species (20, 21, 83). Moreover, we showed that EspM and the regulatory substrates, EspE and EspF, are functionally conserved in *M. tuberculosis* (22, 28). Future research will be aimed at defining the ESX-1 transcriptional network in *M. tuberculosis.*

Overall, this study further defined the regulatory network underlying control of the ESX-1 secretion system and demonstrated its importance during infection. For the first time, we have identified an infection-dependent transcriptional activator responsible for regulating both the ESX-1 components and substrates. Our study will serve as a foundation for understanding the molecular complexities of ESX-1 regulation in the host. We are now poised to define the molecular mechanisms underlying how the ESX-1 system senses and responds to a changing host environment.

## MATERIALS AND METHODS

Bacterial strains were derived from the *M. marinum* M parental strain (ATCC BAA-535) and were maintained as previously described (20, 22, 28, 84). Nomenclature follows the conventions proposed by Bitter et al. (34). Genetic deletions were performed using allelic exchange as previously described (20, 22, 28, 84, 85). Hemolytic activity was measured against sRBCs as described previously (20, 22, 28, 84). Cell-associated and secreted mycobacterial proteins were isolated and analyzed as described in references (28, 84). Protein levels were measured using Western blot analysis. RNA extraction was performed from *M. marinum* using the Qiagen RNeasy kit, followed by qRT-PCR relative to the levels of *sigA* as described previously (22, 44). Macrophage (RAW 264.7) cytotoxicity was measured using ethidium homodimer uptake following infection by *M. marinum* as described in references (39, 84). Mycobacterial CFUs were obtained similarly to reference (28). Protein modeling was performed using Robetta and Pfam as indicated. Zebrafish larvae infections with *M. marinum* were performed, and bacterial burden was measured using fluorescent pixel counts as reference (42). Statistical analysis was performed using GraphPad Prism v.9 or R within the latest version of R Studio IDE. Detailed methods are in the supplemental material.

## AFTER EPUB

*[This article was published on 6 March 2024 with errors in the affiliation for Rachel M. Cronin. The affiliations were corrected in the current version, posted on 12 March 2024.]*

## SUPPLEMENTAL MATERIAL

Data Set S1 mbio.03357-23-s0001.xlsx Proteomics data.

Data Set S2 mbio.03357-23-s0002.xlsx Data related to whole genome sequencing of mycobacterial strains.

Fig. S1 mbio.03357-23-s0003.pdf Genotypic confirmation of *espN* deletion and complementation*.*

Fig. S2 mbio.03357-23-s0004.pdf EspN and ESX-1 substrate expression in deletion and overexpression *M. marinum strains.*

Fig. S3 mbio.03357-23-s0005.pdf ESX-1 membrane complex transcripts are regulated by ESX-1.

Fig. S4 mbio.03357-23-s0006.pdf EspM and EspN do not regulate each other transcriptionally in *M. marinum* under laboratory conditions.

Fig. S5 mbio.03357-23-s0007.pdf Expression of EspM_NT_ impacts ESX-1 gene expression.

Supplemental material mbio.03357-23-s0008.pdf Supplemental legends and methods.

Table S1 mbio.03357-23-s0009.pdf Strains and plasmids.

Table S2 mbio.03357-23-s0010.pdf Primers used in this study.

ASM does not own the copyrights to Supplemental Material that may be linked to, or accessed through, an article. The authors have granted ASM a non-exclusive, world-wide license to publish the Supplemental Material files. Please contact the corresponding author directly for reuse.
