# Supplementary figures and images for: The antagonistic transcription factors, EspM and EspN, regulate the ESX-1 secretion system in M. marinum
Source: mBio. 2024 Mar 6;15(4):e03357-23. doi: 10.1128/mbio.03357-23 (PMC11005418; doi:10.1128/mbio.03357-23)

A.

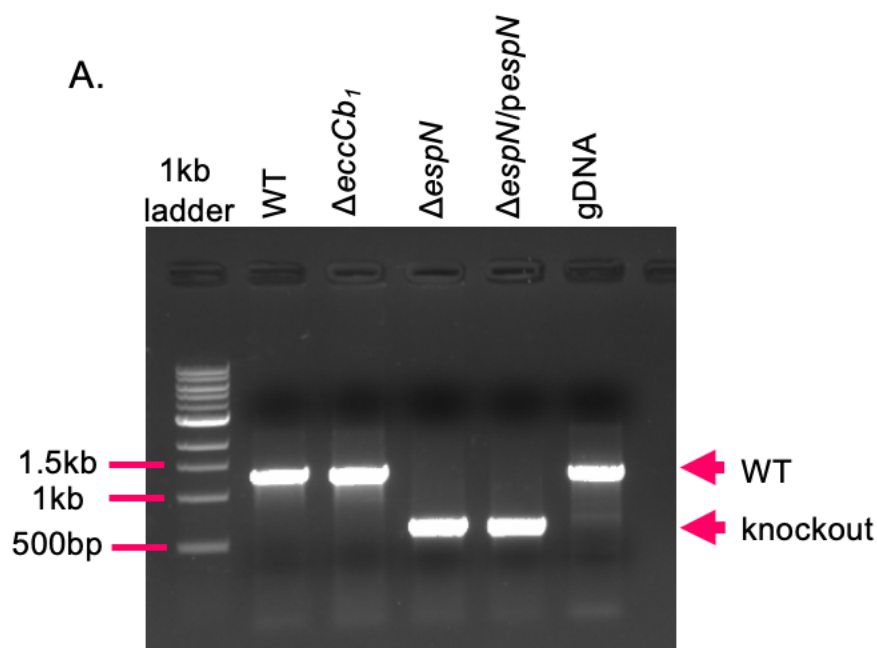

B.

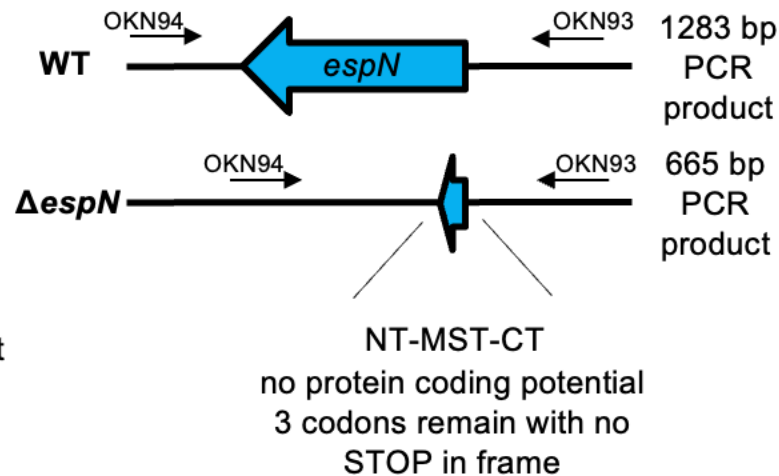

C.

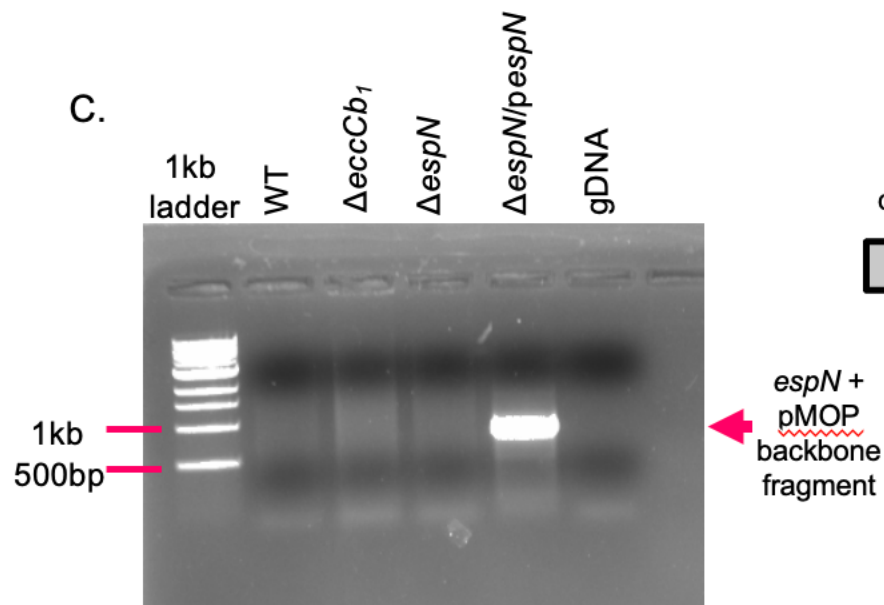

D.

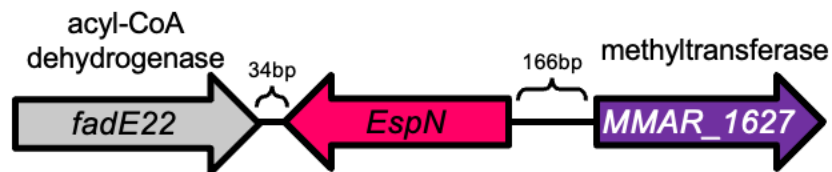

Supplement: Fig. S1 — Genotypic confirmation of espN deletion and complementation. [file mbio.03357-23-s0003.pdf]

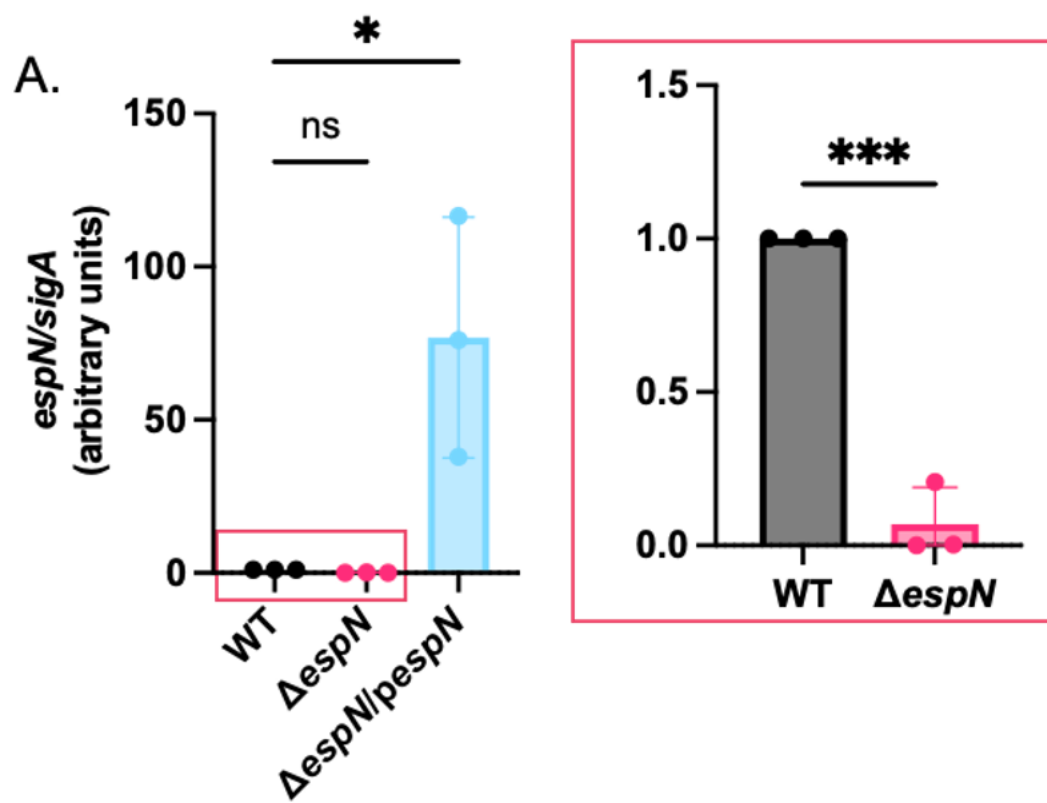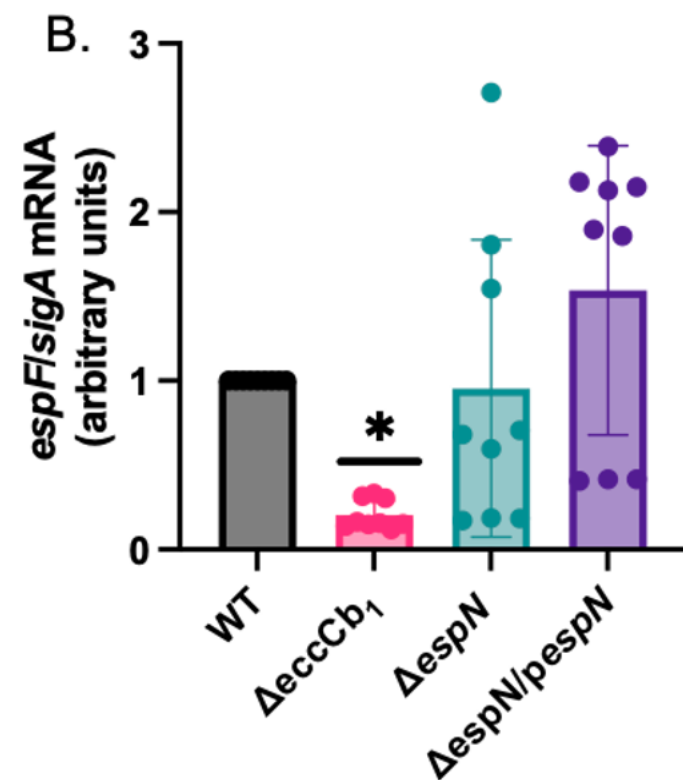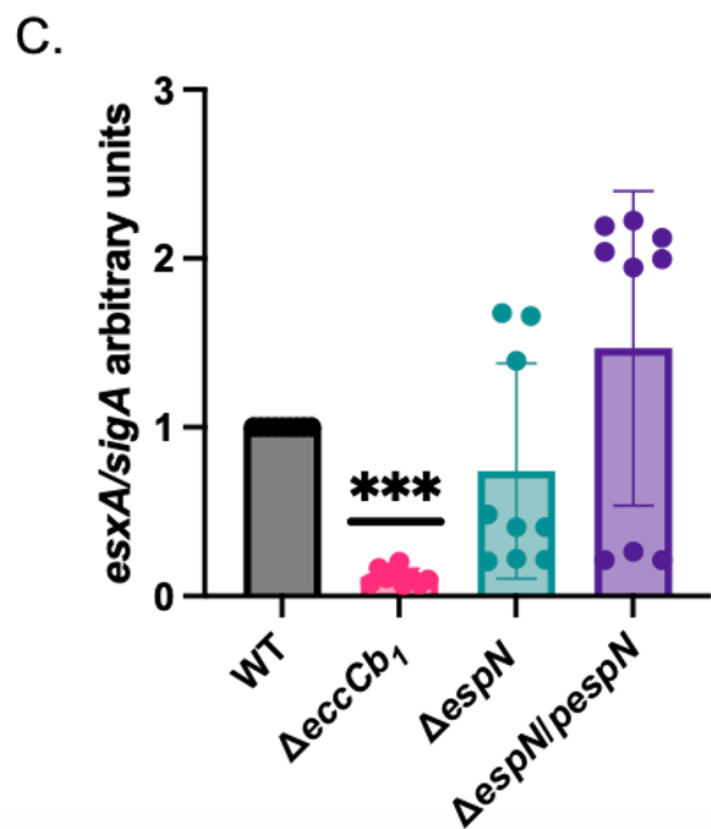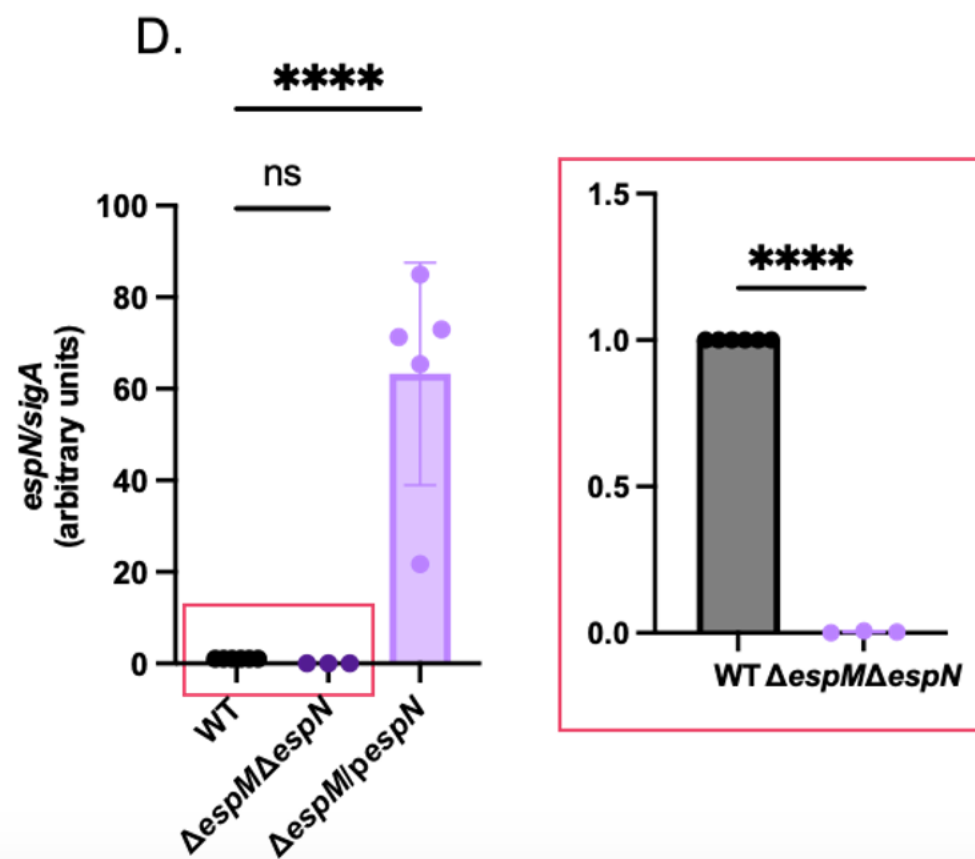

Supplement: Fig. S2 — EspN and ESX-1 substrate expression in deletion and overexpression M. marinum strains. [file mbio.03357-23-s0004.pdf]

A

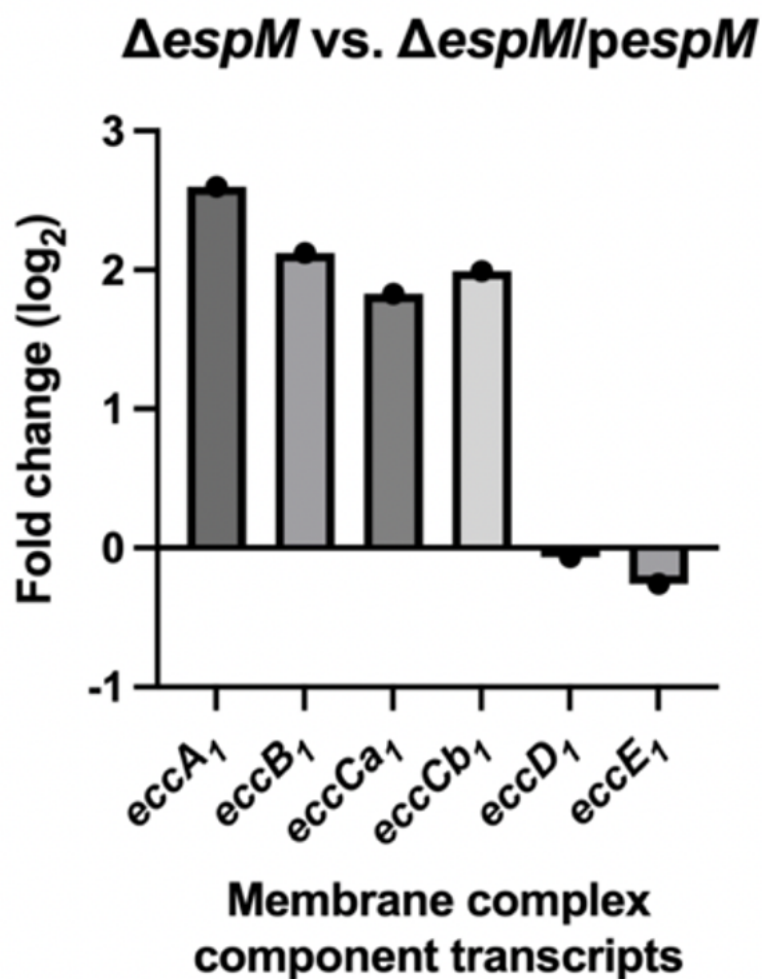

B

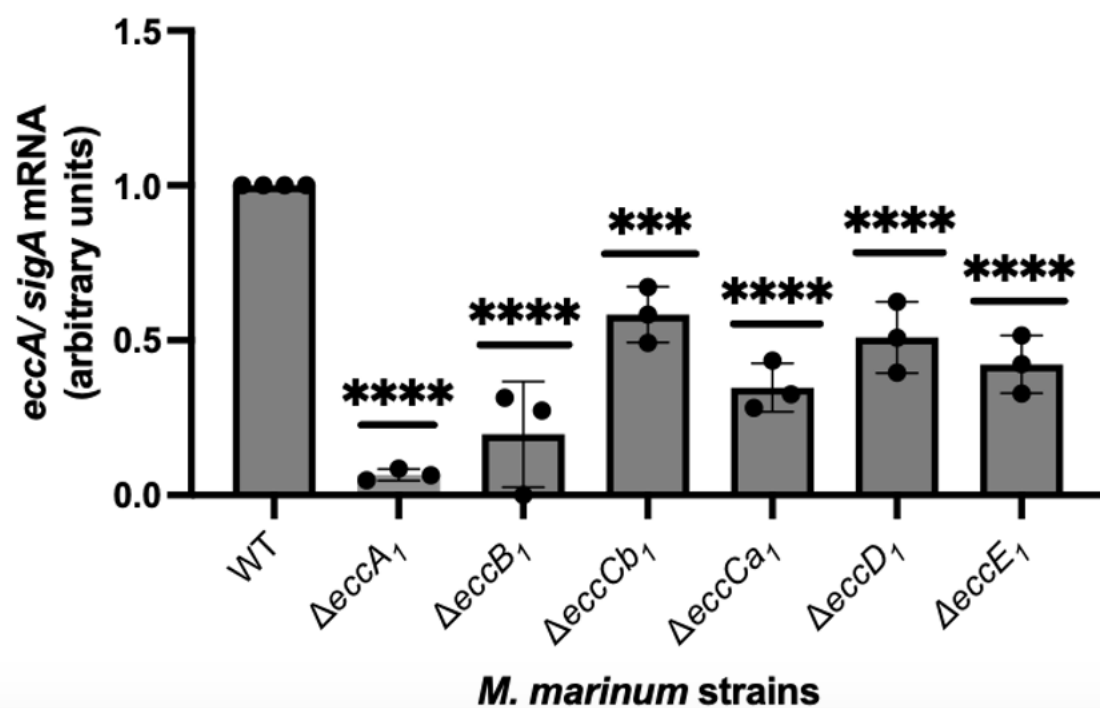

Supplement: Fig. S3 — ESX-1 membrane complex transcripts are regulated by ESX-1. [file mbio.03357-23-s0005.pdf]

A

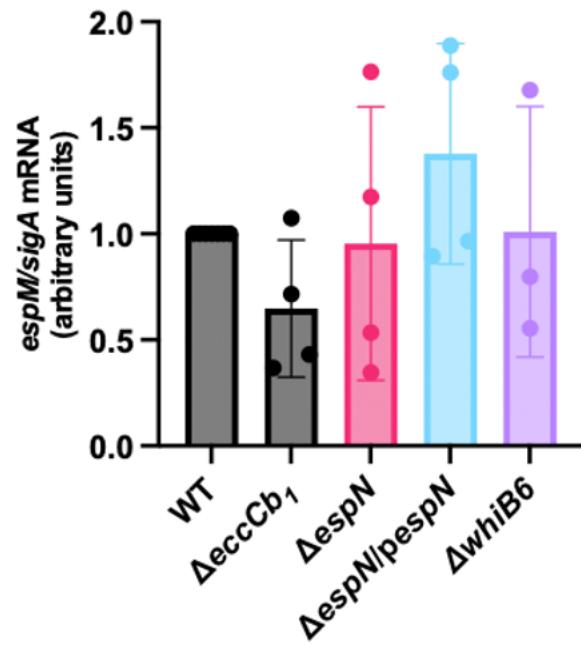

B

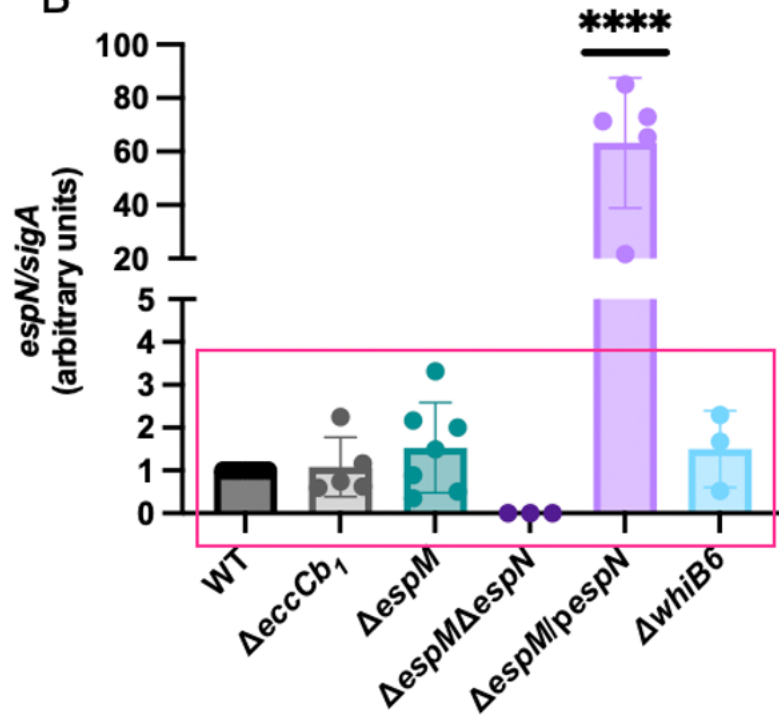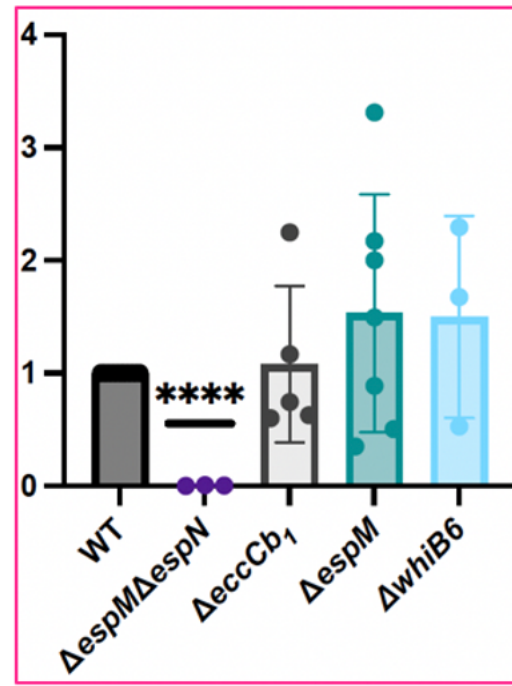

Supplement: Fig. S4 — EspM and EspN do not regulate each other transcriptionally in M. marinum under laboratory conditions. [file mbio.03357-23-s0006.pdf]

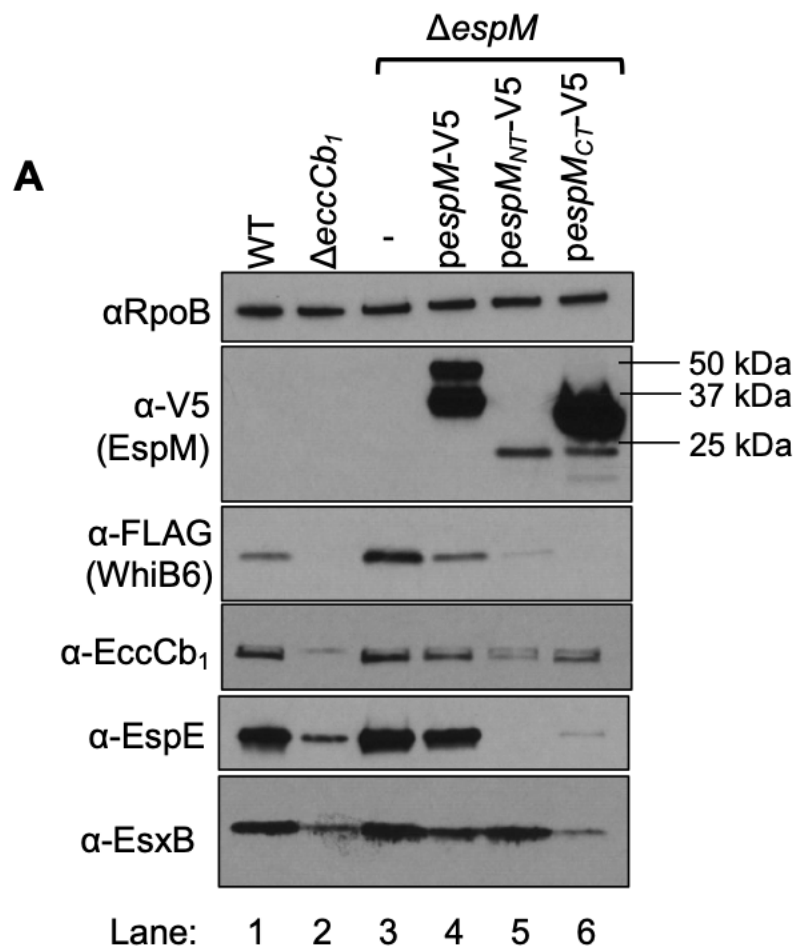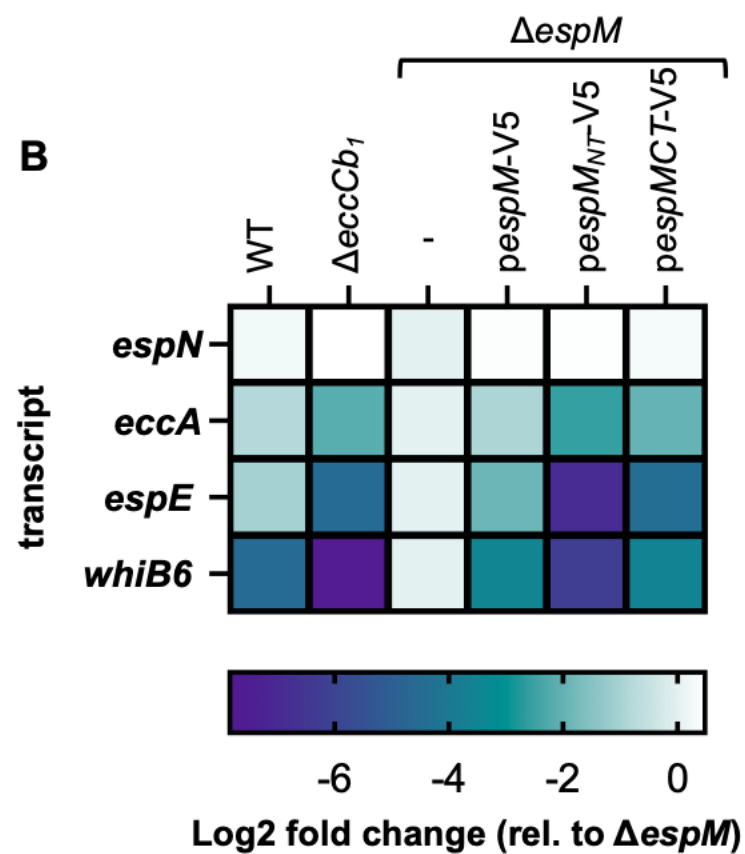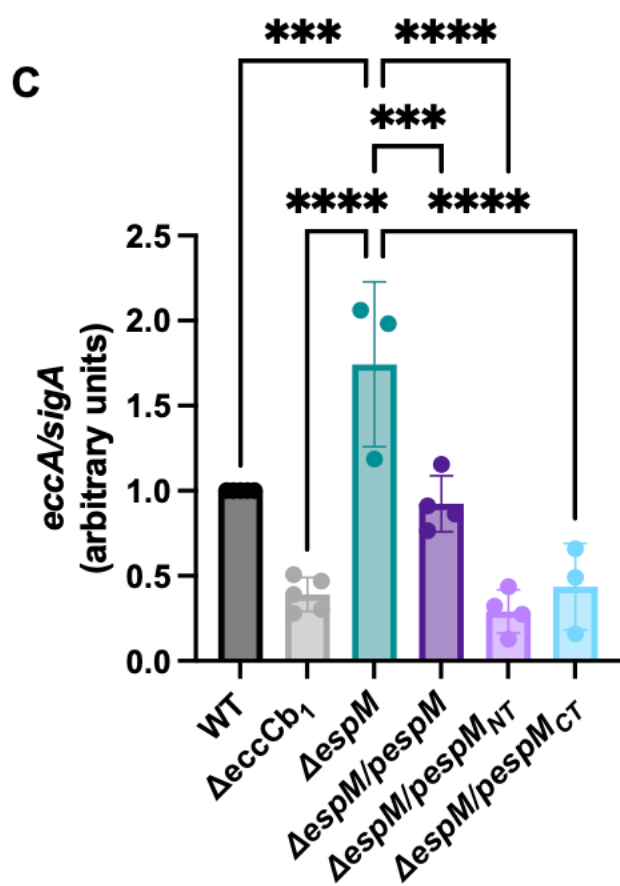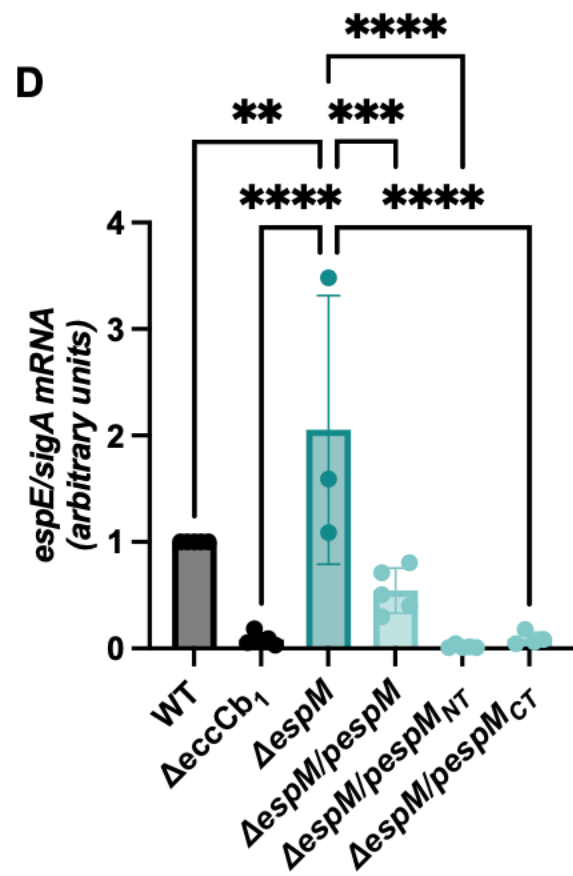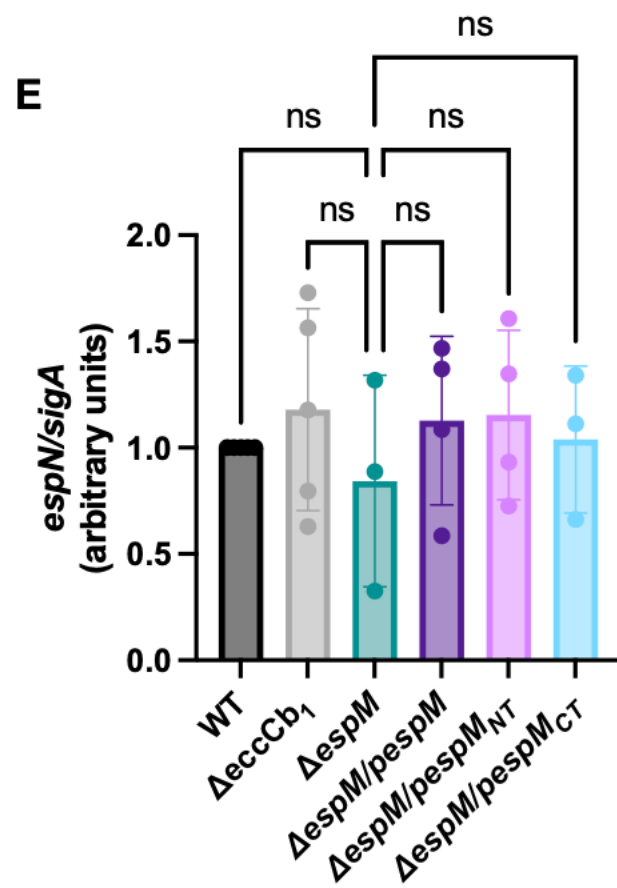

Supplement: Fig. S5 — Expression of EspMNT impacts ESX-1 gene expression. [file mbio.03357-23-s0007.pdf]
