# Supplementary material for: The antagonistic transcription factors, EspM and EspN, regulate the ESX-1 secretion system in M. marinum
Source: mBio. 2024 Mar 6;15(4):e03357-23. doi: 10.1128/mbio.03357-23 (PMC11005418; doi:10.1128/mbio.03357-23)
Supplement: Supplemental material — Supplemental legends and methods. [file mbio.03357-23-s0008.pdf]

Supplemental Legends:

Fig. S1. Genotypic confirmation of *espN* deletion and complementation. A. PCR of upstream and downstream of the *espN* gene using OKN93 and OKN94 primers to confirm genetic deletion in *M. marinum* strains. WT = 1283 bp; Δ*espN* = 665 bp. *M. marinum* genomic DNA (gDNA) serves as a negative control. **B.** Schematic of the *espN* deletion using allelic exchange. **C.** PCR of p*espN* plasmid in *M. marinum* strains using OKN139 and MOPF primers. A 775 bp band indicates the p*espN* is present. *M. marinum* gDNA serves as a negative control. **D.** Schematic of the genetic locus including *espN* in *M. marinum.*

Fig. S2. EspN and ESX-1 substrate expression in deletion and overexpression *M. marinum* strains. A. Relative qRT analysis of *espN* in *M. marinum* strains compared to *sigA* transcript levels. Statistical analysis was performed using a one way-ordinary ANOVA (*P*=.0092), followed by a Dunnett's multiple comparison test. * *P*=.0114. Inset: Zoom in of the comparison between the WT and Δ*espN* strain. The levels of *espN* were compared using an unpaired student's t-test. *** *P*=.0002. B. *espF* in *M. marinum* strains compared to *sigA* transcript levels. Statistical analysis was performed using a one way-ordinary ANOVA (*P*=.0008), followed by a Dunnett's multiple comparison's test. * *P*=0.0264 and C. *esxA* in *M. marinum* strains compared to *sigA* transcript levels. Statistical analysis was performed using a one way-ordinary ANOVA (*P*=.0002), followed by a Dunnett's multiple comparison's test. ** *P*=0.0063. D. Relative qRT analysis of *espN* in *M. marinum* strains compared to *sigA* transcript levels. Statistical analysis was performed using a one way-ordinary ANOVA (*P*<.0001), followed by a Dunnett's multiple comparison's test. ****

P<0.0001. Inset: Comparison between the WT and Δ*espM*Δ*espN* strains. The levels of *espN* were compared using an unpaired student's t-test. **** *P*<0.0001.

Fig. S3. ESX-1 membrane complex transcripts are regulated by ESX-1. A. Log_2 fold-change of ESX-1 membrane complex component transcripts from RNA sequencing data published in Sanchez et al (22). The *eccA-eccCb*$_1$ transcripts were significantly upregulated in the Δ*espM* strain compared to the overexpression strain (*P = 1.07e-23, 1.11e-19, 3.99e-15, 2.13e-16*). The *eccD*$_1$ and *eccE*$_1$ transcripts were not significantly different between the two strains by RNA sequencing. **B.** Relative qRT analysis of *eccA* transcript in *M. marinum* strains compared to *sigA* transcript levels. Statistical analysis was performed using a one way-ordinary ANOVA *(P*=.0001), followed by a Tukey's multiple comparison test. *** *P*=.0005, **** *P*<.0001.

Fig. S4: EspM and EspN do not regulate each other transcriptionally in *M. marinum* under laboratory conditions. qRT PCR on total RNA extracted from *M. marinum* following growth *in vitro* measuring **A.** *espM* transcript or **B.** *espN* transcript relative to *sigA*. None of the measured changes of the *espM* transcript in panel A are significantly different from the WT strain based on a one-way ordinary ANOVA. In panel B, significance was determined using a one-way ordinary ANOVA (*P<.0001*), followed by a Dunnett's multiple comparison test. **** *P*<.0001 compared to the WT strain. In the inset, the Δ*espM*/*pespN* strain was excluded. In the inset, an unpaired t-test was performed between the WT and the Δ*espM*Δ*espN* and WT strains (*P*<.0001).

Fig. S5: Expression of EspM$_{NT}$ impacts ESX-1 gene expression. A) Western blot of *M. marinum* whole cell lysates. All strains include a whiB6-Fl allele. EspM complementation strains are tagged at the C-terminus with a V5 tag. RpoB is a loading control. EspE and

EsxB are ESX-1 substrates. B) Relative qRT-PCR analysis of M. *marinum* strains compared to *sigA* transcript levels. The values were normalized to those in the Δ*espM* strain and plotted as the log2 fold change rela-tive to the Δ*espM* strain as a heat map. The qRT-PCR data plotted in this heat map include at least three independent biological replicates each in technical triplicate, are shown in Fig. 5D, and panels C) *eccA*, D) *espE,* and E) *espN* relative to *sigA* in this figure. Each data point is the average of three technical replicates. Statistical analysis was performed using a one-way ordinary ANOVA followed by a Dunnett's post-hoc test vs the Δ*espM* strain. C) ANOVA *P*<.0001, *** (vs WT, *P*=.0005, vs Δ*espM/pespM, P*=.0003), **** *P* <.0001, D) ANOVA *P*<.0001, ** *P*=.0068, *** *P*=.0002, **** *P* <.0001, E) ANOVA *P* value was not significant.

Table S1. Strains and Plasmids used in this study.

Table S2. List of Primers used in this study.

Dataset S1. Proteomics Dataset. The dataset is reprocessed data from Ref. (22).

DatasetS2. Genomics Dataset. The dataset includes whole genome sequencing of *M. marinum* strains.

Supporting Methods

Growth and Generation of Bacterial Strains Mycobacterial strains were derived from the *M. marinum* M parental strain ("WT", ATCC BAA-535). Parental strains including a 3X-FLAG epitope at the C-terminus of WhiB6 are denoted by "W6Fl" and were used for WhiB6 protein detection (1). All bacterial strains were maintained as described previously, and are listed in Table S1 (1-4) . All liquid cultures of *M. marinum* strains were grown in Middlebrook 7H9 (Sigma Aldrich, St. Louis, MO) broth with 0.5% glycerol and 0.1% Tween-80 unless otherwise noted at 30°C. For growth on agar plates, *M. marinum* strains were struck for isolation on Middlebrook 7H11 (Sigma Aldrich, St. Louis, MO) agar supplemented with 0.5% glycerol and 0.5% glucose. Broth or agar were supplemented with 20µg/ml kanamycin (IBI Scientific, Dubuque, IA) or 50µg/ml hygromycin (Sigma-Aldrich) as needed. Strains containing integrating plasmids were grown in the absence of antibiotics in liquid media. All assays used the following estimation: 1 $OD_{600}=7.7x10^7$ cells/ml for *M. marinum*. *E. coli* strains were grown in LB (Luria-Bertani) media (VWR) with 50 µg/ml kanamycin, 200 µg/ml hygromycin, 200 µg/ml ampicillin, or 12 µg/ml tetracycline (Thermo Fisher, Waltham, MA) when needed. Cloning and plasmid propagation was performed using DH5α *E. coli* (New England Biolabs, Ipswich, MA). *E. coli* strains were grown at 37°C.

Nomenclature. Nomenclature in this work follows the convention set by Bitter et al. (5). ESX-1 conserved components are denoted Ecc. ESX-1 associated proteins are denoted Esp. Genes labeled with a subscript 1 are associated with the ESX-1 system.

Generation of mycobacterial strains. Oligonucleotide primers were purchased from Integrated DNA Technologies (IDT, Coralville, IA). Plasmids were generated using FastCloning using either *M. marinum* M or *M. tuberculosis* Erdman genomic DNA. Plasmids were introduced into *M. marinum* using electroporation as described previously (1-4, 6, 7). Strains and plasmids are listed in Supplementary Tables S1. All plasmids and genetic deletions were confirmed by targeted DNA sequencing performed by the Notre Dame Genomics and Bioinformatics Facility. Whole plasmid sequencing was performed by Plasmidsaurus (SNPsaurus, LLC).

Whole Genome Sequencing. Genomic DNA was extracted from each *M. marinum* strain as follows: *M marinum* was grown in 25mL of 7H9 media. Ten milliliters of this culture was collected at 3000 rpm for 10 minutes and resuspended in 450μL of bug lysis solution (25mM Tris-HCl pH 7.9, 10mM EDTA, 50mM glucose) and 0.5mg lysozyme (Sigma). This mixture was incubated at 37°C overnight. Following incubation, 100uL of 10% SDS (Fisher) and 0.5mg proteinase K (Sigma) were added and the solution was incubated at 55°C for 30 minutes. Then, 200μL of 5M sodium chloride (Fisher) and 160μL Cetrimide Saline Solution [4.1% sodium chloride, 10% Cetrimide (Sigma)] was added, and the mixture incubated at 65°C for 10 minutes followed by chloroform extraction. The gDNA pellet was washed with isopropanol followed by 70% ethanol and resuspended in TE or EA buffer. Whole genome sequencing (WGS) was performed by SeqCoast Genomics (Short read whole genome sequencing, Illumina with variant calling).

Allelic Exchange. Allelic exchange was performed as previously published to generate mutant *M. marinum* strains (1-4, 7). Briefly, approximately 1,500 base pairs upstream and downstream of the annotated open reading frame (8) was amplified using PCR with the Phusion polymerase. The PCR amplified upstream and downstream regions were introduced into the PCR amplified p2NIL vector [Addgene plasmid number 20188; a gift from Tanya Parish (9) by three-part FastCloning (10)]. Following transformation into DH5α *E. coli* (NEB), colonies were picked from LB plates containing kanamycin (50μg/ml). Plasmids were confirmed using restriction enzyme digestion with enzymes chosen depending on the insert composition. Next, confirmed p2NIL plasmids were digested with PacI (NEB), treated with Antarctic Phosphatase (NEB), and ligated with the pGOAL19 vector (Addgene plasmid number 20190; a gift from Tanya Parish (9), as previously described (1-4). Following confirmation by restriction digestion with PacI and AflII (NEB) and targeted DNA sequencing at the Notre Dame Genomics and Bioinformatics Facility, plasmids were quantified using a Nanodrop (Thermo Fisher). 2 μg of the confirmed plasmid was irradiated with 0.1 J/cm2 of UV light using a CL-1000 UV crosslinker (UVP). Electrocompetent *M. marinum* cells were transformed using electroporation. 500 μl of *M. marinum* competent cells were electroporated in a Gene Pulser XCell (Bio-Rad) in the presence of 2μg of the UV irradiated plasmid. Transformed *M. marinum* cells were allowed to recover overnight in 2ml of 7H9 media with 0.1% Tween-80. Following overnight recovery, cells were pelleted by centrifugation, resuspended in 200 μl of medium, and plated on 7H11 agar (Sigma) supplemented with oleic acid-albumin-dextrose-catalase

(OADC), 50 µg/ml hygromycin (Corning), and 60 µg/ml 5-bromo-4-chloro-3-indolyl-β-D-galactopyranoside (X-gal) with 2% sucrose (Macron).

Hemolysis Assays. Sheep red blood cell (sRBC) assays were performed as previously described (1-4). Briefly, *M. marinum* strains were grown in 7H9 medium with 0.1% Tween-80. Bacteria were grown to an OD_{600} between $2.0x10^8$ cells and $6.5x10^8$ cells. $5.0x10^8$ bacteria were washed three times with 1X phosphate buffered saline (PBS) and incubated with sRBCs (Hardy Diagnostics, Santa Maria, CA) at 30°C for 2h. OD_{405} readings were obtained in technical triplicate using a SpectraMax plate reader.

Protein preparation and analysis: ESX-1 secretion assays. ESX-1 protein secretion assays were performed exactly as previously described (2, 11). *M. marinum* was cultured in 5 ml of Middlebrook 7H9 defined broth, (Sigma-Aldrich, St. Louis, MO) + 0.1% Tween-80 (Fisher Scientific, Pittsburgh, PA) for three days, then moved to 25 ml Middlebrook 7H9 + 0.1% Tween-80 for two days. *M. marinum* strains were diluted to an OD_{600} of 0.8 in Sauton's broth + 0.01% Tween-80. Following 48 hours of growth at 30°C, *M. marinum* cells were collected by centrifugation. The cells were lysed using a Biospec Mini-BeadBeater-24, and the lysate was clarified by centrifugation. The resulting proteins are the cell-associated protein fraction. Phenylmethylsulfonyl fluoride (PMSF, Roche) was added to the supernatants to a 0.1% final concentration. The supernatant was then filtered using 0.2 µm Nalgene Stericups with polyethersulfone (PES) filters to remove extraneous bacteria. Supernatants were concentrated by ultrafiltration using a 3,000-molecular-weight-cutoff (MWCO) Amicon filter (Millipore, Sartorius),

yielding the secreted protein fraction. Pellets were resuspended in 1X PBS + 0.1% PMSF (Roche) and bead beaten using a Biospec Mini-BeadBeater-24 and silica beads. Pellets were clarified by centrifugation and transferred to new tubes. Mycobacterial cell-associated protein fractions were quantified using a Micro BCA Protein Assay Kit (ThermoFisher).

Western Blotting. All SDS-PAGE gels were loaded with 10μg of protein unless otherwise indicated. For mycobacterial protein samples, all SDS-PAGE gels were 4-20% gradients (BioRad). All antibodies were diluted in 5% nonfat dry milk in 1X PBS + 0.1% Tween-20. Rpoβ (anti-RNA polymerase beta mouse monoclonal antibody [clone: 8RB13]; VWR) was diluted 1:20,000. The following reagents were obtained through BEI resources, NIAID, NIH: polyclonal anti-*Mycobacterium tuberculosis* CFP-10 (gene Rv3874; antiserum, rabbit; NR-13801) and polyclonal anti-*Mycobacterium tuberculosis* Mpt-32 (gene Rv1860; antiserum, rabbit; NR-13807). CFP-10 was used at a 1:5,000 dilution. Mpt-32 was used at a 1:30,000 dilution. The EspE antibody (1:5,000 dilution) was obtained from Frederic Carlsson (12). $EccCb_1$ was detected using a custom rabbit polyclonal antibody against the CDKQEFPSSEFKVKR peptide (Genscript). The $EccCb_1$ antibody was used at a 1:5,000 dilution. The C-terminally tagged WhiB6 protein was detected using a monoclonal α-FLAG M2 antibody (Millipore) at a 1:5,000 dilution. The C-terminally tagged EspM protein was detected using a mouse monoclonal α-V5 antibody (Sigma) at a 1:5,000 dilution. Horse radish peroxidase (HRP)-conjugated goat α-mouse immunoglobulin secondary antibody (Bio-Rad) was used at a 1:5000 dilution to detect the α-EsxA, α-RNAP, α-FLAG, and α-V5 antibodies.

Horse radish peroxidase (HRP)-conjugated goat α-rabbit immunoglobulin secondary antibody (Bio-Rad) was used at a 1:20,000 dilution to detect the α-Mpt32 and α-EsxB antibodies. Horse radish peroxidase (HRP)-conjugated goat α-rabbit immunoglobulin secondary antibody (Bio-Rad) was used at a 1:5,000 dilution to detect α-EspE. All proteins were detected using the LumiGLO chemiluminescent substrate kit (SeraCare, Milford, MA) and X-ray film (RPI, Mt. Prospect, IL).

RNA Extraction. *M. marinum* was cultured in 5 ml of Middlebrook 7H9 media (Sigma Aldrich) + 0.1% Tween-80 (Fisher Scientific, Pittsburgh, PA) for three days, then moved to 25 ml Middlebrook 7H9 + 0.1% Tween-80 for two days. *M. marinum* strains were diluted to OD_{600}=0.8 in Sauton's broth + 0.01% Tween-80 and grown for 48h at 30°C. Bacterial culture pellets were collected by centrifuging 15 ml of Sauton's culture. These pellets were frozen. Thawed bacterial pellets were resuspended Qiagen RLT buffer (Qiagen, Hilden, Germany) supplemented with 1% β-mercaptoethanol. Lysates were generated by bead beating pellet resuspension 3 times for 30 seconds using silica beads and a Biospec Mini-BeadBeater-16 (BioSpec Products Inc., Batesville, OH, USA). Total RNA was extracted from clarified lysates using the RNeasy Mini Kit (Qiagen), according to manufacturer's instructions.

qRT-PCR. 500 ng-1 µg of RNA was treated with Promega RQ1 DNase (Promega) according to manufacturer instructions and supplemented with 5mM $MgCl_2$ and 10mM $CaCl_2$. 1µl of DNase treated RNA was converted to cDNA using random hexamers (IDT) and Superscript II (SSII) Reverse Transcriptase

(Invitrogen) according to manufacturer's instructions. cDNA was quantified using a NanoDrop 2000 (Thermo Fisher).

qRT-PCR reactions were prepared using 250ng of cDNA mixed with SYBR Select Master Mix (Applied Biosystems, Carlsbad, CA) and 1μM of each oligonucleotide (unless noted otherwise). *sigA* was used as a housekeeping reference gene control and was detected using oligonucleotide primers sigA-F/sigA-R. All other oligonucleotide primers are listed in Supplementary Table S2. All qRT-PCR reactions were run using Applied Biosystems MicroAmp Fast 96 well plates (0.1mL, Life Technologies). All plates were run on a QuantStudio 3 Real-Time PCR System (Thermo Fisher). Cycle conditions were as follows: 50°C for 2 min., 95°C for 10 min; 40 cycles at 95°C for 15 sec and 60°C for 1 min; a dissociation step of 95°C for 15 sec., 60°C for 1 min., 95°C for 15 sec., and 60°C for 15 sec.

All qRT-PCR reactions were analyzed using ΔΔ Ct comparisons. All qRT-PCR results were normalized to WT transcript abundance using the following equations:

$$\Delta Ct = Ct(\text{gene of interest}) - Ct(\text{housekeeping gene})$$

Then:

$$\Delta\Delta Ct = \Delta Ct(\text{treated sample}) - \Delta Ct(\text{untreated sample})$$

Then:

$$2^{-\Delta\Delta Ct} = \text{fold change}$$

Macrophage Cytotoxicity. RAW264.7 murine macrophages (ATCC TIB-71) were grown in high glucose, high pyruvate Dulbecco's Modified Eagle's Medium (DMEM) (Gibco, Dublin, Ireland) with 10% heat-inactivated fetal bovine serum (FBS) (Avantor, Radnor, PA). All RAW264.7 cells were cultured at 37°C under 5% CO_2. Macrophages were washed with sterile phosphate buffered saline (PBS) pH 7.4 (Gibco, Dublin, Ireland) and passaged using cell scraping as needed.

Macrophage infections were performed as described previously (2, 13). Briefly, macrophage monolayers were seeded in 1ml DMEM + 10% FBS at $3x10^5$ in 24-well plates (Greiner Bio-One, Germany). 24h after seeding, RAW264.7 cells were infected in technical triplicate with mycobacterial strains at an MOI=4, where 1 OD_{600} = $7.7x10^7$ cells. At 2 hpi, each well was treated with 100 µg/ml Gentamicin (RPI Corporation, Mt. Prospect, IL). Macrophage monolayers were washed 3X with sterile 1X PBS 6 hpi and fresh DMEM + 10% FBS was added. To assay cytotoxicity, culture media was removed 24 hpi and 250µl of EthD-1 (1µl/ml) + Calcein-AM (0.25µl/ml) (Live/Dead Viability/Cytotoxicity Kit; Life Technologies, Carlsbad, CA) solution in 1X PBS was added. Cells were incubated for 30 min at 37°C + 5% CO_2 and imaged using a Zeiss AxioObserver A1 inverted microscope with phase-contrast, rhodamine (red), and green fluorescent protein (GFP) filters. Ten images were obtained per well, and dead cells were quantified using ImageJ as described previously (13).

RNA isolation from Macrophage Infections. RNA isolation was modified from the method described by Rohde et al. (14). RAW 264.7 cells (ATCC TIB-71) were seeded in 35 mL DMEM plus 10% FBS per plate at 500 x 10^5 cells/plate in

143 cm^2 tissue culture dishes (Fisher Scientific) and allowed to grow for 24 h. Before infection, bacteria were de-clumped by 15 passages through a 27-gauge needle (EXELINT). Bacteria were added at an estimated MOI of 20 and mixed. Infections were incubated at 37°C + 5% CO_2 for 4 h. Infections were harvested at 4 hours post-infection (hpi). Medium was aspirated and intracellular bacteria were stabilized and isolated using guanidine thiocyanate-based lysis buffer (4 M guanidine thiocyanate, 0.5% Na N-lauryl sarcosine, 25 mM sodium citrate, and 0.1 M β-mercaptoethanol) as described here (15). Samples were transferred to a 50 mL conical tube (Corning) and vortexed for 2 min. Samples were passed through a 23-gauge needle (Becton-Dickinson) 14 times. Samples were centrifuged at 4000 rpm for 45 min and the bacteria pellets were recovered. Pellets were re-suspended in 1 mL lysis buffer and re-pelleted through centrifugation at 12,500 rpm for 5 min. Bacteria samples were washed with 1 mL phosphate-buffered saline (PBS) with 0.1% Tween 20. Pelleted bacteria were digested with 5 mg/mL lysozyme for 15 min at room-temperature. Trizol heated to 65°C was added to each tube and bacteria were lysed with 0.1 mm zirconia disruption bead (RPI) and by three 30 second pulses on a mini BeadBeater (Biospec Products). Total RNA was isolated by chloroform extraction followed by addition of ethanol and direct application to Qiagen RNeasy column purification.

CFU Assays. RAW 264.7 macrophages were seeded in 1ml DMEM plus 10% FBS per well at $5x10^5$ cells/ml in a 24-well plate (Greiner Bio-One, Germany) and allowed to grow for 24h at 37°C + 5% CO_2. Bacteria were added at an MOI of 0.2 ($1x10^5$ cells/ml) in technical triplicate and mixed. Infections proceeded for 2h

at 37°C + 5% CO_2. 2-hour post-infection (2hpi) entry assays were harvested. For harvesting, medium was removed and 0.5ml of sterile lysis buffer (H2O plus 0.1% [vol/vol] Tween 80) was added. Plates were then incubated at 37°C + 5% CO_2 for 10min before scraping the wells and pipetting up and down. Cells were diluted at 1:1000 using sterilized dilution buffer (1x PBS plus 0.05% [vol/vol] Tween 80). 50µl of the dilutions were plated on Middlebrook 7H11 plates supplemented with 10% (vol/vol) oleic acid-albumin-dextrose-catalase (OADC) and 0.5% (vol/vol) glycerol. For uninfected controls, 50µl of undiluted cells were plated. Each technical triplicate was plated in duplicate (6 plates/time point). For 24, 48, 72, and 96hpi time points, gentamycin (RPI Corporation, Mt. Prospect, IL) was added at 100µg/ml 2hpi. At 4hpi, cells were washed three times with sterile 1x PBS and 1ml fresh medium was added to each well. All time points were harvested exactly as described above. At 48hpi, wells for 72 and 96hpi time points were washed three times with sterile 1x PBS and 1ml fresh medium was added to each well. Colonies were counted following approximately 1 week-incubation at 32°C.

Protein Modeling and Alignments. Protein modeling was performed using Robetta, Pfam, and Alphafold as indicated (16-18). Protein sequences were obtained from Mycobrowser or Biocyc, where appropriate (8, 19).

Zebrafish infections. Zebrafish larvae were anaesthetized and infected at 2 days post-fertilization with 150-200 colony forming units (CFU) of each strain and assessed at 1-day post-infection and 5 days post-infection for bacterial burden using fluorescent pixel counts (20), a validated longitudinal readout of bacterial burden. 1-day post infection readouts controls for starting levels of bacterial

burden. Fluorescence is enumerated over the area of the infected fish by calculating the number of pixels above background using a constant threshold. These results were analyzed using R 4.2.2 within the latest version of RStudio IDE using in-house workflows. Fold change was the ratio of fluorescence of *ΔespN* compared to WT. Graphing was similarly performed in R using ggplot2. All zebrafish husbandry and experimental procedures were performed in accordance and compliance with policies approved by the Duke University Institutional Animal Care and Use Committee (protocol A091-20-04).

Statistical Analysis. All statistical analyses were performed in GraphPad Prism version 9 or R 4.2.2 within the latest version of RStudio IDE. Statistical significance was determined by performing an ordinary one-way or two-way ANOVA followed by either a Dunnett's, Tukey's, or Sidak's multiple comparisons test. P-values for individual experiments are noted in the figure legend and in the text. All experiments were conducted on at least three biological replicates, each with technical triplicates where available.

Supplementary References

1. R. E. Bosserman *et al.*, WhiB6 regulation of ESX-1 gene expression is controlled by a negative feedback loop in Mycobacterium marinum. *Proc Natl Acad Sci U S A* 10.1073/pnas.1710167114 (2017).
2. R. E. Bosserman, K. R. Nicholson, M. M. Champion, P. A. Champion, A New ESX-1 Substrate in Mycobacterium marinum That Is Required for Hemolysis but Not Host Cell Lysis. *J Bacteriol* **201** (2019).
3. K. G. Sanchez *et al.*, EspM Is a Conserved Transcription Factor That Regulates Gene Expression in Response to the ESX-1 System. *mBio* **11** (2020).
4. A. E. Chirakos, K. R. Nicholson, A. Huffman, P. A. Champion, Conserved ESX-1 substrates EspE and EspF are virulence factors that regulate gene expression. *Infect Immun* 10.1128/IAI.00289-20 (2020).
5. W. Bitter *et al.*, Systematic genetic nomenclature for type VII secretion systems. *PLoS Pathog* **5**, e1000507 (2009).
6. T. Parish, Electroporation of Mycobacteria. *Methods Mol Biol* **2314**, 273-284 (2021).
7. R. E. Bosserman, C. R. Thompson, K. R. Nicholson, P. A. Champion, Esx Paralogs Are Functionally Equivalent to ESX-1 Proteins but Are Dispensable for Virulence in Mycobacterium marinum. *J Bacteriol* **200**, e00726-00717 (2018).
8. A. Kapopoulou, J. M. Lew, S. T. Cole, The MycoBrowser portal: a comprehensive and manually annotated resource for mycobacterial genomes. *Tuberculosis (Edinb)* **91**, 8-13 (2011).
9. T. Parish, N. G. Stoker, Use of a flexible cassette method to generate a double unmarked Mycobacterium tuberculosis tlyA plcABC mutant by gene replacement. *Microbiology* **146 (Pt 8)**, 1969-1975 (2000).
10. C. Li *et al.*, FastCloning: a highly simplified, purification-free, sequence- and ligation-independent PCR cloning method. *BMC Biotechnol* **11**, 92 (2011).
11. A. E. Chirakos, K. R. Nicholson, A. Huffman, P. A. Champion, Conserved ESX-1 Substrates EspE and EspF Are Virulence Factors That Regulate Gene Expression. *Infect Immun* **88** (2020).
12. F. Carlsson, S. A. Joshi, L. Rangell, E. J. Brown, Polar localization of virulence-related Esx-1 secretion in mycobacteria. *PLoS Pathog* **5**, e1000285 (2009).
13. E. A. Williams *et al.*, A Nonsense Mutation in Mycobacterium marinum That Is Suppressible by a Novel Mechanism. *Infect Immun* **85** (2017).
14. K. H. Rohde, R. B. Abramovitch, D. G. Russell, Mycobacterium tuberculosis invasion of macrophages: linking bacterial gene expression to environmental cues. *Cell Host Microbe* **2**, 352-364 (2007).
15. P. D. Butcher, J. A. Mangan, I. M. Monahan, Intracellular gene expression. Analysis of RNA from mycobacteria in macrophages using RT-PCR. *Methods Mol Biol* **101**, 285-306 (1998).
16. M. Baek *et al.*, Accurate prediction of protein structures and interactions using a three-track neural network. *Science* **373**, 871-876 (2021).
17. J. Jumper *et al.*, Highly accurate protein structure prediction with AlphaFold. *Nature* **596**, 583-589 (2021).
18. R. D. Finn *et al.*, Pfam: the protein families database. *Nucleic Acids Res* **42**, D222-230 (2014).
19. P. D. Karp *et al.*, The BioCyc collection of microbial genomes and metabolic pathways. *Brief Bioinform* **20**, 1085-1093 (2019).
20. K. Takaki, C. L. Cosma, M. A. Troll, L. Ramakrishnan, An in vivo platform for rapid high-throughput antitubercular drug discovery. *Cell Rep* **2**, 175-184 (2012).
21. J. W. Saelens *et al.*, An ancestral mycobacterial effector promotes dissemination of infection. *Cell* https://doi.org/10.1016/j.cell.2022.10.019 (2022).
22. K. G. Sanchez, R. J. Prest, K. R. Nicholson, K. V. Korotkov, P. A. Champion, Functional Analysis of EspM, an ESX-1-Associated Transcription Factor in Mycobacterium marinum. *J Bacteriol* 10.1128/jb.00233-22, e0023322 (2022).
