# Supplementary material for: The antagonistic transcription factors, EspM and EspN, regulate the ESX-1 secretion system in M. marinum
Source: mBio. 2024 Mar 6;15(4):e03357-23. doi: 10.1128/mbio.03357-23 (PMC11005418; doi:10.1128/mbio.03357-23)
Supplement: Table S1 — Strains and plasmids. [file mbio.03357-23-s0009.pdf]

Table S1. Strains and plasmids used in this study.

Name	Genotype	Reference
**Strains**		
M. marinum M strain	Wild type; parent for all strains	ATCC BAA-535
Δ*whiB6*	M with deletion of the *whiB6* (*MMAR_5437) gene*	(1)
whiB6-FL	M with *whiB6* allele tagged with 3X-FLAG at C-terminus, at *whiB6* locus	(1)
whiB6-FLΔ$eccCb_1$	*whiB6*-FL with deletion of the M with deletion of the $eccCb_1$ *(MMAR_5446)* gene	(1)
whiB6-FLΔ*espN*	*whiB6*-FL with deletion of the M with deletion of the *espN (MMAR_1626)* gene	This study
whiB6-FLΔ*espM*	*whiB6*-FL with deletion of the M with deletion of the *espM (MMAR_5438)* gene	(3)
whiB6-FLΔ*espM*Δ*espN*	*whiB6*-FLΔ*espM* with deletion of the *espN (MMAR_1626)* gene	This study
**Plasmids**		
p2NIL	kan^R, amp^R, *oriE*; Parental vector for allelic exchange	(9)
pGOAL19	amp^R, GOAL cassette includes *hygR*, *lacZ*, *sacB*, *oriE*; Parental vector for allelic exchange	(9)
p2NILΔ*espN*GOAL	*M. marinum espN* flanking regions. kan^R, hyg^R, *lacZ, sacB*	This study
p*espN*	*espN* from *M. marinum* behind the pMOP promoter, hyg^R. *attB* integration	This study
pMSP12*mCerulean*	*mCerulean* gene expressed behind the pMSP12 promoter. kan^R. Episomal.	(21)
p*espM*-V5	*espM (MMAR_5438)* from *M. marinum* behind the pMOP promoter, hyg^R. *attB* integration. V5 C-terminal tag.	(22)
p$espM_{NT}$-V5	Amino acids 1-133 from *espM (MMAR_5438)* from *M. marinum* behind the pMOP promoter, hyg^R. *attB* integration. V5 C-terminal tag.	(22)
p$espM_{CT}$-V5	Amino acids 127-363 from *espM (MMAR_5438)* from *M. marinum* behind the pMOP promoter, hyg^R. *attB* integration. V5 C-terminal tag.	This study
p*espN* -7A	*espN* from *M. marinum* behind the pMOP promoter with a -7T>A mutation, hyg^R. *attB* integration.	This study
p*espN* -7C	*espN* from *M. marinum* behind the pMOP promoter with a -7T>C mutation, hyg^R. *attB* integration.	This study
p*espN* -7G	*espN* from *M. marinum* behind the pMOP promoter with a -7T>G mutation, hyg^R. *attB* integration	This study
p*espN* -12A	*espN* from *M. marinum* behind the pMOP promoter with a -12T>A mutation, hyg^R. *attB* integration	This study

p*espN* -12C	*espN* from *M. marinum* behind the pMOP promoter with a -12T>C mutation, *hyg*^{R}. *attB* integration	This study
p*espN* -12G	*espN* from *M. marinum* behind the pMOP promoter with a -12T>G mutation, *hyg*^{R}. *attB* integration.	This study
p*espE*$_{TB}$	*espE* from *M. tuberculosis* behind the pMSP promoter, *hyg*^{R}. *attB* integration	(4)
p*espF*	*espF* from *M. marinum* behind the pMSP promoter, *hyg*^{R}. *attB* integration	(4)
