# Supplementary material for: The antagonistic transcription factors, EspM and EspN, regulate the ESX-1 secretion system in M. marinum
Source: mBio. 2024 Mar 6;15(4):e03357-23. doi: 10.1128/mbio.03357-23 (PMC11005418; doi:10.1128/mbio.03357-23)
Supplement: Table S2 — Primers used in this study. [file mbio.03357-23-s0010.pdf]

Table S2: List of oligonucleotide primers used in this study.

Name	Sequence 5' → 3'	Application and Reference
OKN88	cgtggtgtcacgctcgtGACATCACCGGGCTCAACAAC	Primer pair (A&B, upstream arm) to delete *MMAR_1626* using p2NIL. This study.
OKN89	CGGGCCTTAAGATGTGGACATCGCGCAAGACCC	
OKN90	CCACATCTTAAGGCCCGCGAGGCGGATGAC	Primer pair (C&D, downstream arm) to delete *MMAR_1626* using p2NIL. This study.
OKN91	gcagtcaggcaccgtATCGGATTCACCTGGGAGCATGAC	
OKN93	TATCGCCACGTTGCCAGATCC	Δ*MMAR_1626* F and R genotyping primers This study.
OKN94	TCAAGCTGGCCGAACACATGG	
OKN138	aggagtccagccatTGCGCGATGTCCACATACAGC	F and R primers to generate *MMAR_1626* insert for FastCloning into pMOP. This study.
OKN139	gcctgagcggtcccgactagtATCCGCCTCGCGGGCTCAG	
sigA-F	TCGAGGTGATCAACAAGCTG	*sigA* qRT primers (13)
sigA-R	TGGATCTCCAGCACCTTCTC	
olc 208	GACGGCGTCTACAAGGTCTG	*espE* qRT primers (4)
olc 209	CCGGAATGTTCGGGAGTAGG	
ORS225	AGATTCCGCTGGGCGTTTGC	*whiB6* qRT primers (1)
ORS226	TCTGCCAGCGACCGAAGTTG	
5438FqRT	CGTCACCAACAGCCCAAACG	*espM* qRT primers (3)
5438RqRT	CTGCGCTGACTGATGTCGAG	
ORS152	GCCTTCGTCAGTGAGTTTCC	*eccCb*$_1$ qRT primers (1)
ORS153	TGGGCTGCGATTTGAGCTAC	
OKNq24	TTGAAGGATCCGTCCTACCG	*eccA* qRT primers This study.
OKNq25	CGAGTTCTTCTTGGGCTTCG	
OKNq46	CCACATACAGCCAGTTCTGC	*espN* (*MMAR_1626*) qRT primers This study.
OKNq47	GCCTTGGTCAACGACTTGAG	
-7AF	CGGCTCGTATAAAGTGTGGAATTGT	F and R primers to introduce a -7T>A mutation to the Mycobacterial Optimal Promoter. This study.
-7AR	ACA ATT CCA CAC TTT ATA CGA GCC G	
-7CF	CGGCTCGTATAACGTGTGGAATTGT	F and R primers to introduce a -7T>C mutation to the Mycobacterial Optimal Promoter. This study.
-7CR	ACA ATT CCA CAC GTT ATA CGA GCC G	
-7GF	CGGCTCGTATAAGGTGTGGAATTGT	F and R primers to introduce a -7T>G mutation to the Mycobacterial Optimal Promoter. This study.
-7GR	ACA ATT CCA CAC CTT ATA CGA GCC G	
-12AF	GCTTCCGGCTCGAATAATGTGTGGA	

-12AR	TCC ACA CAT TAT TCG AGC CGG AAG C	F and R primers to introduce a -12T>A mutation to the Mycobacterial Optimal Promoter. This study.
-12CF	GCTTCCGGCTCGCATAATGT GTGGA	F and R primers to introduce a -12T>C mutation to the Mycobacterial Optimal Promoter. This study.
-12CR	TCC ACA CAT TAT GCG AGC CGG AAG C	
-12GF	GCTTCCGGCTCGGATAATGT GTGG	F and R primers to introduce a -12T>G mutation to the Mycobacterial Optimal Promoter. This study.
-12GR	TCC ACA CAT TAT CCG AGC CGG AAG C	
